# Clustering Performance Analysis Using Chaotic and Lévy Flight-Enhanced Black-Winged Kite Algorithms

**DOI:** 10.3390/biomimetics11030200

**Published:** 2026-03-09

**Authors:** Taybe Alabed, Sema Servi

**Affiliations:** Department of Computer Engineering, Faculty of Technology, Selcuk University, Konya 42130, Türkiye; 248273002006@ogr.selcuk.edu.tr

**Keywords:** clustering, Black Winged Kite Algorithm (BKA), logistic map, Levy flight, optimization algorithms

## Abstract

Clustering is a fundamental unsupervised learning technique used to uncover hidden patterns in unlabeled data. Although metaheuristic algorithms have demonstrated effectiveness in clustering, many suffer from premature convergence and limited population diversity. This study employs the Black-Winged Kite Algorithm (BKA) and its enhanced variants, Chaotic BKA (CBKA), Lévy Flight-based BKA (LBKA), and Chaotic Levy BKA (CLBKA), to address these limitations in centroid-based clustering formulated as a Sum of Squared Errors (SSE) minimization problem. Chaotic logistic mapping improves search diversity and adaptability, while Levy flight introduces long-range exploration. In addition, Cauchy based perturbations are incorporated to enhance convergence stability. The algorithms are evaluated on sixteen UCI benchmark datasets, with 30 independent runs conducted under different population and iteration settings. Experimental results show that CLBKA consistently achieves superior clustering performance in terms of accuracy and stability. Statistical validation using the Friedman and Wilcoxon tests confirms significant performance differences, with CLBKA obtaining the lowest mean rank across configurations. The findings indicate that integrating chaotic dynamics and Levy flight mechanisms enhances clustering robustness and optimization efficiency.

## 1. Introduction

Clustering has evolved from early conceptual investigations of grouping phenomena in the social and behavioral sciences into a formalized statistical and computational framework. Initially concerned with identifying latent structures within qualitative observations, clustering gradually became a central analytical tool in quantitative data analysis. This evolution led to the development of classical algorithmic paradigms such as centroid-based, hierarchical, and density-based approaches. In recent years, the focus has expanded toward density-aware and dynamic clustering models capable of handling complex, high-dimensional, and irregular data structures [1,2]. Clustering is a core unsupervised learning approach that aims to organize data samples into meaningful groups based on similarity, while simultaneously enhancing within-cluster compactness and between-cluster separability. Owing to its ability to reveal hidden structures in unlabeled data, clustering has become an essential component in various fields such as pattern recognition, machine learning, data mining, and signal processing [3,4,5]. Since clustering problems can be naturally expressed as continuous optimization tasks, metaheuristic optimization techniques have attracted increasing interest due to their effectiveness in navigating complex, nonlinear, and multimodal search spaces and avoiding poor local solutions [6]. Accordingly, a wide range of nature-inspired metaheuristic algorithms, including Genetic Algorithms (GA), Particle Swarm Optimization (PSO), Grey Wolf Optimizer (GWO), and Ant Lion Optimizer (ALO), have been successfully adapted to clustering by iteratively refining cluster centroids using population-based search strategies [7]. Nevertheless, despite their promising performance, many metaheuristic clustering algorithms are still prone to premature convergence, reduced population diversity, and stagnation in local optima, particularly when applied to high-dimensional or noise-contaminated datasets [8].

The Black-Winged Kite Algorithm (BKA) is a metaheuristic algorithm proposed in 2024 [9], inspired by the hovering, predatory, and migratory behaviors of the black-winged kite bird. The algorithm incorporates key behavioral mechanisms, including attack-based movement patterns, Cauchy-driven migration, and adaptive leader selection, to regulate the balance between exploration and exploitation during the search process. Although BKA has shown competitive results on standard benchmark optimization problems, it may still encounter challenges such as early convergence and limited ability to escape locally optimal regions [10]. To alleviate such shortcomings, chaotic mapping strategies have been widely employed in metaheuristic algorithms, as they exhibit sensitivity to initial conditions, ergodic behavior, and the capability to generate non-periodic and diverse search trajectories [11]. Among various chaotic systems, the logistic map has been frequently reported as an effective mechanism for maintaining population diversity and mitigating stagnation phenomena in evolutionary search processes [12]. In addition, Lévy flight-based search strategies have been increasingly integrated into metaheuristic frameworks due to their heavy-tailed step-length distributions, which enable occasional long-distance movements and significantly enhance global exploration, thereby improving the ability of optimization algorithms to escape local optima and explore broader regions of the search space [13].

Although the Black-Winged Kite Algorithm exhibits an effective balance between exploration and exploitation, its performance may deteriorate in later iterations due to premature convergence and a gradual loss of population diversity. To address these limitations, three enhanced variants of BKA have been developed. The Chaotic Black-Winged Kite Algorithm (CBKA) incorporates chaotic logistic mapping to increase search diversity, while the Lévy Flight-based Black-Winged Kite Algorithm (LBKA) introduces Lévy flight-driven transitions to strengthen global exploration. Furthermore, the hybrid Chaotic Lévy Black-Winged Kite Algorithm (CLBKA) combines both chaotic mapping and Lévy flight mechanisms to achieve a more effective and balanced exploration strategy. These enhanced BKA variants have demonstrated strong performance on standard benchmark functions as well as real-world engineering optimization problems [10]. However, despite their promising optimization capabilities, their applicability to data clustering problems has not yet been comprehensively investigated.

In this study, the BKA, CBKA, LBKA, and CLBKA are employed to address data clustering by formulating it as a global optimization problem. Unlike conventional clustering techniques, the proposed approach does not rely on prior assumptions regarding data distribution or cluster shape. Instead, cluster formation is achieved through a metaheuristic-driven search process that aims to identify optimal cluster centroids by minimizing a predefined clustering objective function. The goal of this study is to address premature convergence and limited exploration in metaheuristic-based clustering by developing chaotic and Lévy flight-enhanced variants of the Black-Winged Kite Algorithm. Unlike existing chaotic or Lévy flight-enhanced clustering algorithms, this study introduces a unified and phase-aware integration of chaotic control, Lévy flight exploration, and Cauchy mutation within the Black-Winged Kite framework, specifically tailored for centroid-based clustering.

The main contributions of this study can be summarized as follows. First, the original Black-Winged Kite Algorithm and its enhanced variants (CBKA, LBKA, and CLBKA) are adapted to the clustering domain through a centroid-based optimization framework. The remainder of this paper is organized as follows. Section 2 presents a comprehensive review of related work in metaheuristic-based clustering. Section 3 describes the methodological framework, including the original Black Winged Kite Algorithm and its enhanced variants, CBKA, LBKA, and CLBKA, together with their mathematical foundations and integration mechanisms. The clustering formulation, benchmark datasets, statistical evaluation methods, and time complexity analysis are also detailed in this section. Section 4 provides the experimental results and discussion, including comparative performance analysis, literature-based evaluations, and statistical significance tests such as the Friedman, Nemenyi, and Wilcoxon procedures, as well as sensitivity analysis of key parameters. Finally, Section 5 concludes the paper with key findings and directions for future research.

## 2. Related Work

Prior to the emergence of metaheuristic clustering methods, classical algorithms such as K-means [4,14], hierarchical clustering [3], and DBSCAN [15] served as foundational tools in unsupervised learning. These approaches remain widely used due to their simplicity, computational efficiency, and interpretability, and they have played a pivotal role in numerous early clustering applications. However, despite their practical advantages, classical methods often rely on strong assumptions about data distribution, such as spherical clusters in K-means or fixed density thresholds in DBSCAN. As a result, they can face challenges when applied to noisy, non-convex, or high-dimensional datasets [16]. For example, K-means is sensitive to initial centroid selection and may converge to local minima, while DBSCAN may struggle with clusters of varying densities. These limitations have motivated the exploration of alternative clustering strategies that can provide more flexible, global search capabilities. In this context, metaheuristic optimization algorithms have gained increasing attention for their ability to overcome local traps, maintain diversity, and adapt to complex search spaces without strong assumptions about data geometry [17,18].

Building upon this motivation, metaheuristic algorithms have emerged as a powerful alternative for clustering, reformulating it as a global optimization problem. Metaheuristic algorithms have become a dominant paradigm in data clustering by reformulating clustering as a global optimization problem that minimizes intra-cluster distance while maximizing inter-cluster separation. Unlike classical clustering techniques, these approaches provide flexible search mechanisms capable of escaping local optima and reducing sensitivity to initialization. Survey studies consistently report that swarm intelligence and evolutionary algorithms such as Genetic Algorithms (GA), Particle Swarm Optimization (PSO), and Ant Colony Optimization (ACO) outperform traditional clustering approaches across diverse datasets [19,20]. Overall, recent research trends indicate a shift from classical deterministic clustering toward adaptive population-based optimization frameworks that emphasize exploration–exploitation balance and robustness against complex search landscapes.

Several metaheuristic algorithms have been successfully adapted to clustering tasks. For example, Grey Wolf Optimizer (GWO)-based clustering demonstrates competitive performance compared with k-means and fuzzy c-means [21], while history-driven Artificial Bee Colony (Hd-ABC) algorithms enhance convergence stability [22]. Similarly, Whale Optimization Algorithm (WOA), Chimp Optimization Algorithm (ChOA), Tree Seed Algorithm (TSA), and Artificial Algae Algorithm (AAA) have achieved promising clustering results in terms of accuracy and robustness [23,24,25,26,27]. Despite these advancements, many studies reveal recurring methodological limitations, including premature convergence, loss of population diversity, and performance degradation in high-dimensional or noisy datasets. These limitations have motivated the development of hybrid and enhanced metaheuristic frameworks.

Hybridization has emerged as a major research direction, where complementary search strategies are combined to improve convergence and stability. The Genetic Black Hole (GBH) algorithm, for instance, integrates global exploration with intensive local exploitation to achieve faster convergence and higher clustering accuracy [28]. Early foundational works on ACO- and ABC-based clustering established the effectiveness of cooperative swarm behaviors for centroid optimization and laid the groundwork for subsequent hybrid metaheuristic designs [29,30]. A key overarching trend in recent literature is the increasing reliance on hybrid mechanisms to address exploration–exploitation imbalance, suggesting that single-strategy algorithms may be insufficient for complex clustering landscapes.

Chaotic maps have gained significant attention as diversity-enhancing mechanisms in metaheuristic clustering. Logistic and other chaotic mappings introduce ergodic, non-periodic search behaviors that improve exploration capability and mitigate stagnation [31,32]. Studies integrating chaotic dynamics into algorithms such as BKA, PSO, Fox Optimization, Bee Colony Optimization, and ACO consistently report improvements in convergence speed and solution quality [33,34,35,36,37,38]. However, existing chaotic approaches often focus primarily on parameter perturbation rather than structural search adaptation, leaving open challenges regarding scalability and adaptive control in dynamic or high-dimensional clustering scenarios.

Another prominent enhancement strategy involves Lévy flight, which introduces long-range stochastic movements to improve global exploration. Originating from Cuckoo Search, Lévy-based search dynamics have demonstrated superior performance in escaping local optima and exploring large search spaces [39,40]. Lévy-enhanced WOA, PSO–K-means hybrids, and Black Hole-based clustering algorithms have shown improved stability and clustering accuracy compared to classical methods [41]. Several studies have directly applied Lévy flight-enhanced metaheuristic algorithms to data clustering problems. Lévy flight-based WOA and hybrid PSO–K-means clustering frameworks have consistently shown superior clustering accuracy, stability, and robustness compared to classical clustering methods and their non-enhanced counterparts, with statistical validation confirming the effectiveness of Lévy flight strategies [42,43,44]. Applications in real-world domains further confirm the effectiveness of Lévy-based exploration strategies [45]. Nevertheless, many Lévy-based methods exhibit sensitivity to parameter settings and may introduce excessive randomness if not carefully balanced with local exploitation mechanisms.

More recently, hybrid strategies combining chaotic dynamics with Lévy flight have attracted increasing interest. Such approaches aim to integrate nonlinear adaptive control with long-range exploration, yielding improved convergence behavior in optimization and clustering tasks [46,47,48,49]. These studies suggest that combining complementary enhancement strategies can address the limitations of single-mechanism metaheuristics. Despite these advances, a systematic integration of chaotic control and Lévy-driven exploration within a unified clustering framework remains relatively underexplored, particularly for newly developed algorithms such as the Black-Winged Kite Algorithm.

Overall, the literature indicates a clear transition toward hybrid and adaptive metaheuristic clustering frameworks, accompanied by the widespread integration of chaotic and Lévy-based mechanisms to enhance exploration and population diversity. Despite these advances, persistent challenges such as premature convergence, scalability limitations, and stability issues across heterogeneous datasets remain unresolved. Motivated by these observations, this study introduces a hybrid Chaotic Lévy Black-Winged Kite Algorithm (CLBKA) to achieve a more balanced and robust clustering optimization strategy.

## 3. Materials and Methods

**Figure 1:**
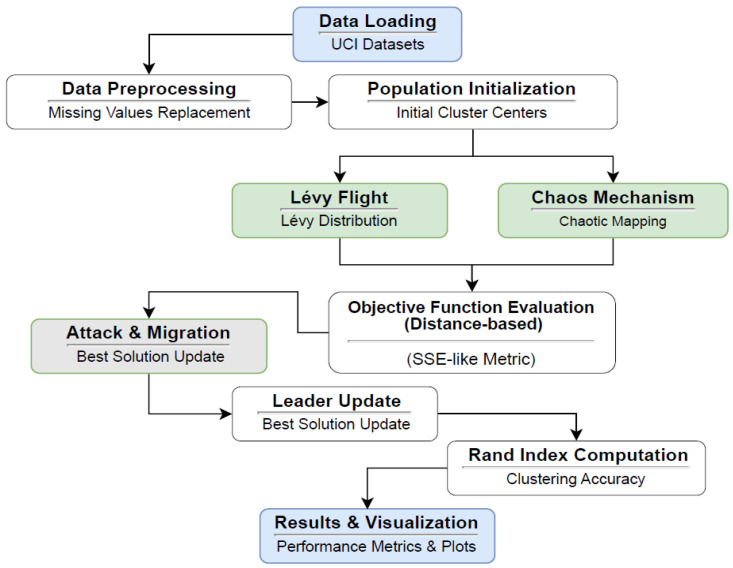
Overview of the clustering procedure.

The methodological design of this study is structured to comprehensively evaluate the clustering performance of the proposed Black-Winged Kite Algorithm (BKA) and its enhanced variants, CBKA, LBKA, and CLBKA. The overall process encompasses a series of systematic stages, including dataset acquisition, preprocessing, initial population generation, fitness evaluation via the sum of squared errors (SSE), and iterative optimization through chaos-induced and Lévy flight-based mechanisms. Each solution undergoes dynamic updates via attack and migration behaviors, with periodic leader selection to guide convergence. Finally, clustering quality is assessed using both internal (SSE) and external (Rand Index) metrics. To facilitate clarity and reproducibility, the entire methodological pipeline is schematically illustrated in Figure 1, which outlines the sequential flow of operations from data input to result visualization.

### 3.1. Black-Winged Kite (BKA) Algorithm

The Black-Winged Kite Algorithm (BKA) is a nature-inspired metaheuristic whose design is motivated by the distinctive hovering capability and efficient hunting strategies of the BKA. These biological characteristics enable the bird to effectively scan large areas, precisely identify targets, and rapidly converge toward prey, which are analogous to global exploration and convergence behaviors in optimization processes. By abstracting the kite’s movement patterns and hunting dynamics into computational rules, BKA provides an efficient mechanism for guiding candidate solutions toward optimal regions within the search space [9].

#### 3.1.1. Attack Behavior

**Figure 2:**
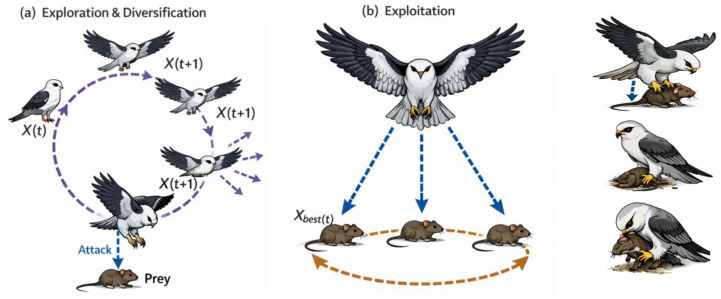
Two hunting behaviors of the black-winged kite: (**a**) pre-attack hovering and (**b**) hovering during prey search.

The black-winged kite employs a highly specialized hunting strategy characterized by stable hovering, continuous observation, and rapid target-oriented descent [50]. As illustrated in Figure 2, the kite remains suspended in midair to assess potential prey and determine the most suitable attack trajectory. Once a target is identified, the bird initiates a swift and controlled descent, capturing its prey with high precision and minimal energy expenditure. This behavior inspires the attack phase of the Black-Winged Kite Algorithm, which emphasizes accuracy and efficient movement toward promising solutions. Mathematically, the attack mechanism of BKA is modeled using the position update rule given in Equation (1), where the control parameter defined in Equation (2) regulates the step size and search intensity during the attack phase:

(1) $$x_{i,j}^{t+1}=\;\left\{\begin{array}{c} x_{i,j}^{t}+n\left(1+sin\left(r\right)\right)\times x_{i,j}^{t}\;p<r\\ x_{i,j}^{t}+n\times \left(2r-1\right)\times x_{i,j}^{t}\;else \end{array}\;\right\}$$

(2) $$n=0.05\times e^{-2\times {\left(\frac{t}{T}\right)}^{2}}$$

In these equations $X_{i,j}^{t}$ and $x_{i,j}^{t+1}$ denote the position of the *i* black-winged kite and the *j* black-winged kite at time *t* and (t+1), respectively. The variable r represents a uniformly distributed random number within the interval [0,1], while P is a predefined constant set to 0.9. The parameter T indicates the maximum number of iterations, and t corresponds to the current iteration count.

#### 3.1.2. Migration Behavior

**Figure 3:**
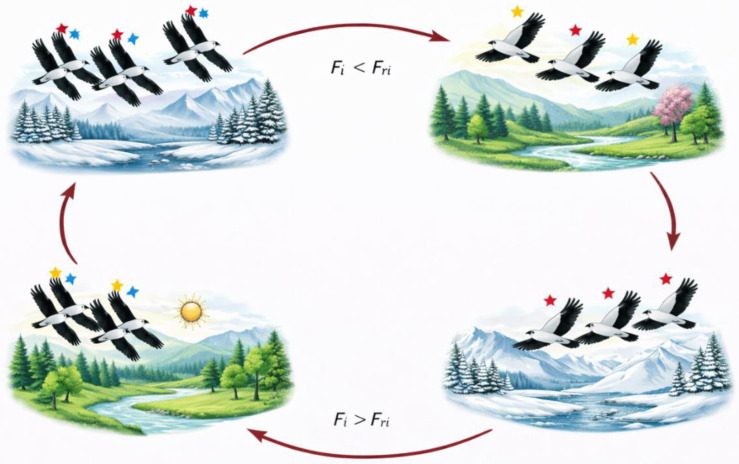
Strategic migration behavior of Black-winged kites in BKA.

The migration mechanism of the BKA is inspired by the collective movement behavior of black-winged kites, which enhances the algorithm’s exploration capability. In natural environments, bird migration is influenced by external factors such as climate conditions and food availability, while dominant individuals play a key role in directing the flock. As illustrated in Figure 3, these leadership-driven dynamics result in adaptive movement patterns during migration. In the BKA framework, this behavior is modeled by associating leadership with solution fitness: when a candidate solution exhibits inferior fitness compared to a randomly selected counterpart, it follows that solution; otherwise, it assumes the leadership role and guides the search process [51].

This strategy enables the dynamic selection of effective leaders, which plays a crucial role in maintaining a successful migration process. The mathematical formulation describing the migration behavior of the black-winged kite within the BKA framework is given in Equation (3), where the control parameter m is defined in Equation (4).

In this formulation $L_{j}^{t}$ denotes the value of the best solution in the $j^{th}$ dimension at iteration t, while $\chi_{i}^{t+1}$ represent the current and updated positions of the $i^{th}$ candidate solution, respectively. The fitness values of the current candidate and the randomly selected solution are represented by $F_{i}\;$ and $F_{ri}$. The term $C\left(0,1\right)\;$ introduces a perturbation based on the Cauchy distribution, which enhances population diversity and prevents premature convergence, as defined in Equation (5) and simplified in Equation (6).

The parameter m, calculated using a sinusoidal function as shown in Equation (4), controls the step size of the migration movement and introduces nonlinearity into the update process. Through the combined effect of fitness-based leadership selection and Cauchy-distributed perturbations, the migration mechanism improves exploration efficiency while preserving the algorithm’s ability to converge toward promising regions of the search space.

(3) $$x_{i,j}^{t+1}=\left\{\begin{array}{c} x_{i,j}^{t}+c\left(0,1\right)\times \left(x_{i,j}^{t}-L_{j}^{t}\right)\;F_{i}<F_{ri}\;\\ x_{i,j}^{t}+c\left(0,1\right)\times \left(L_{t}^{j}-m\times x_{i,j}^{t}\right)\;else \end{array}\right\}$$

(4) $$m=2\times sin(r+\frac{\pi }{2})$$

(5) $$f\left(x,\delta ,\mu \right)=\frac{1}{\pi }\frac{\delta }{\delta^{2}+{\left(x-\mu \right)}^{2^{\prime }}}-\infty <x<\infty$$

When δ=1 and μ=0, the expression for the Cauchy mutation is:

(6) $$f\left(x,\delta ,\mu \right)=\;\frac{1}{\pi }\frac{1}{x^{2}+1^{\prime }}\;-\infty <x<\infty$$

Algorithm 1 presents the pseudocode of BKA, outlining its main steps and core operations in the optimization process [9].
**Algorithm 1.** Pseudo-code of BKA.Algorithm: Black-winged kite algorithm
**Input:** The population size pop, maximum number of iterations T, and variable dimension dim.
**Output:** The best quasi-optimal solution obtained by BKA for a given optimization problem. 1. **Initialization phase** 2. Initialization of the position of Black-winged kites and evaluation of the objective function. 3. Calculate the fitness value of each Black-winged kite 4.      **while** (t < T) **do** 5.    **Attacking behavior**
 6.    **if** p < r 7.     $y_{t+1}^{i,j}$ = $y_{t}^{i,j}$ + n(1 + sin(r)) × $y_{t}^{i,j}$ 8.    **else if do** 9.     $y_{t+1}^{i,j}$ = $y_{t}^{i,j}$ + n × (2r − 1) × $y_{t}^{i,j}$ 10.    **end if**      **Migration behavior** 11.    **if** *F*i < *F*ri do 12.     $y_{t+1}^{i,j}$ = $y_{t}^{i,j}$ + C(0,1) × ($y_{t}^{i,j}$ − $L_{t}^{i}$) 13.     **else if do** 14.     $y_{t+1}^{i,j}$ = $y_{t}^{i,j}$ + C(0,1) × ($L_{t}^{i}$ − m × $y_{t+1}^{i,j}$) 15.    **end if**      **Select the best individual** 16.    **if** $y_{t+1}^{i,j}$ < $L_{t}^{i}$ 17.      Xbest = yᵢⱼ, Fbest = f($y_{t+1}^{i,j}$) 18.     **else if do** 19.      Xbest = $L_{t}^{i}$, Fbest = f($L_{t}^{i}$) 20.     **end if** 21.    **end while** 22. **Return** Xbest and Fbest

### 3.2. Logistic Map

The logistic map is a widely studied one-dimensional discrete-time dynamical system known for exhibiting chaotic behavior, as defined in Equation (7). Although it was originally proposed to describe population growth dynamics, its simple mathematical formulation has led to extensive applications in various domains, including encryption, data security, and nonlinear system modeling [52].

(7) $$x_{k+1}={ax}_{k}\left(1-x_{k}\right),\;$$

where x∈[0,1] denotes the system state at iteration k, while a  represents the control parameter that governs the system’s behavior. Depending on the value of a. The logistic map can exhibit a wide range of dynamical regimes, transitioning from stable fixed points to periodic oscillations and ultimately to fully chaotic behavior.

A key characteristic of the logistic map is its strong sensitivity to initial conditions, which is a fundamental property of chaotic systems. This sensitivity makes the logistic map particularly suitable for applications that require high diversity and unpredictability, such as stochastic optimization and cryptographic processes. In the context of metaheuristic optimization, logistic chaotic sequences are frequently employed to enhance exploration and exploitation by generating non-repetitive pseudo-random patterns that guide the search process. By embedding the logistic map into algorithmic parameters, the search dynamics can be adaptively adjusted, effectively reducing premature convergence and improving overall optimization performance [53].

### 3.3. Lévy Flight

Lévy flight, originating from the studies of mathematician Paul Pierre Lévy, describes a stochastic movement pattern characterized by frequent short steps interrupted by occasional long-distance jumps, governed by a heavy-tailed probability distribution [39]. This type of movement has been empirically observed in various organisms, including birds and fruit flies, whose foraging behaviors exhibit statistical characteristics consistent with Lévy flights. Such naturally occurring patterns have served as inspiration for the design of novel optimization algorithms. In general, Lévy flight is defined by step lengths drawn from a Lévy distribution, resulting in a mixture of localized movements and sporadic long-range transitions. Similar behavioral dynamics have been documented across numerous animal and insect species [54,55]. The mathematical foundations of Lévy flight, established in the early twentieth century, have enabled its later adoption as an effective exploration mechanism within modern metaheuristic optimization frameworks [56]. Lévy flights are broadly recognized as an effective mathematical model for representing the search and movement behaviors of animals and insects. By integrating long-range exploratory steps with short-range exploitative movements, this approach provides a balanced trade-off between exploration and exploitation, which is highly desirable in global optimization algorithms.

A survey of existing studies indicates that Lévy flight has been applied both in its original formulation and through various modified versions. Several adaptations, such as trimmed Lévy flight, smoothed Lévy approaches, and segmented Lévy motion, have been introduced to improve algorithmic efficiency in specific optimization problems [57]. In this study, however, the classical Lévy flight formulation is employed without modification in order to retain the inherent dynamics of the original model.

Lévy flight is classified as a non-Gaussian stochastic process exhibiting heavy-tailed properties, in which movement behavior follows a Lévy stable distribution [58]. This distribution demonstrates power-law characteristics, permitting the occurrence of infrequent yet significant jumps in step length [48]. A simplified expression of the Lévy distribution is presented in Equations (8) and (9).

(8) $$S=\frac{\mu }{{\left|\nu \right|}^{\frac{1}{k}}}$$

for 1 < k ≤ 3, and μ ∼ N(0, $\sigma_{\mu }^{2}$), ν ∼ N(0, $\sigma_{v}^{2}$). μ and ν are random numbers obeying Gaussian distribution, and $\sigma_{\mu }$ and $\sigma_{v}$ satisfy the following equations:

(9) $$\sigma_{\mu }={\left\{\frac{\Gamma \;(1\;+\;k)\;sin(0.5k\pi )}{\Gamma \;[0.5(1\;+\;k)]2^{0.5(k-1)}}\right\}}^{\frac{1}{k}},\;\sigma_{v}=1\;$$

where Γ (·) denotes Gamma function [59].

### 3.4. Description of the Proposed CBKA

In the proposed CBKA framework, the logistic chaotic map is embedded into the original BKA structure to enhance population diversity throughout the search process. As a representative chaotic system, the logistic map produces highly irregular and non-periodic sequences that are extremely sensitive to initial conditions. This inherent unpredictability introduces controlled variations into the search dynamics, thereby increasing diversity among candidate solutions and strengthening the algorithm’s exploratory behavior, particularly in complex and high-dimensional search spaces. In the clustering context, CBKA is adapted to optimize cluster centroids by minimizing the Sum of Squared Errors (SSE) between data points and their nearest centroids. Each individual in the population represents a set of *k* centroids in *d*-dimensional space. During each iteration, centroids are updated using chaos-modulated control parameters, while data points are assigned to the closest centroid based on Euclidean distance. This formulation transforms clustering into a continuous optimization problem suitable for metaheuristic search.

**Figure 4:**
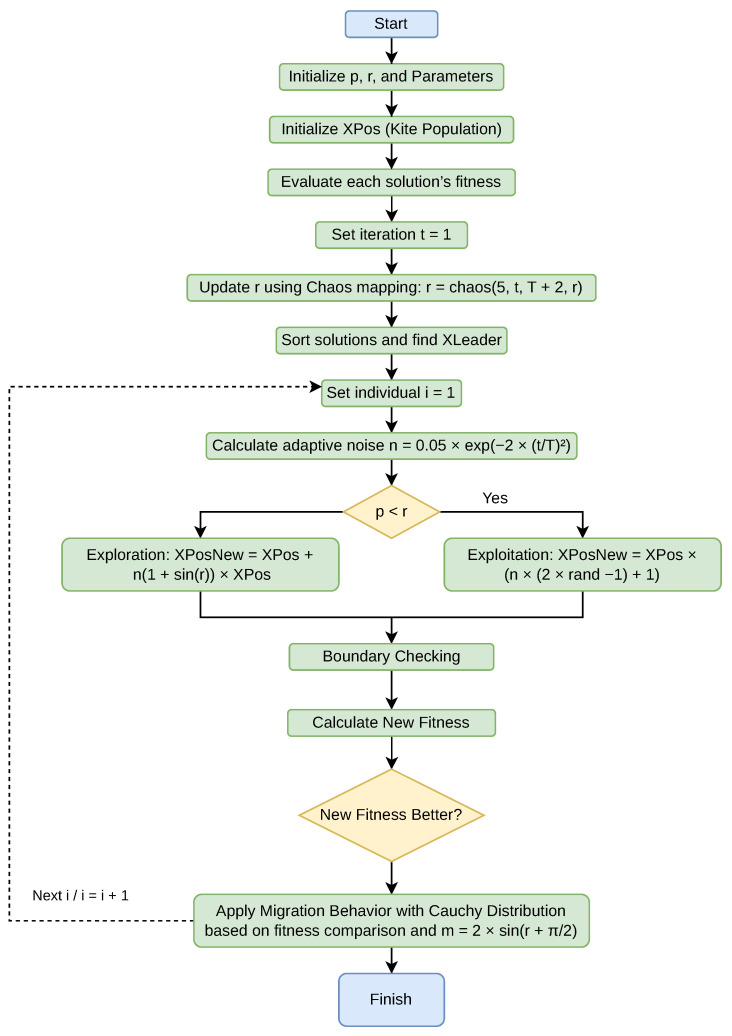
Workflow of the Chaotic Black-Winged Kite Algorithm (CBKA).

By dynamically modulating key control parameters through chaotic sequences, CBKA achieves a more adaptive balance between exploration and exploitation. This mechanism reduces the likelihood of premature convergence by continuously perturbing the search trajectory, allowing the algorithm to escape local optima while maintaining steady convergence toward promising regions. Consequently, the integration of chaotic dynamics enables CBKA to preserve convergence efficiency while improving its ability to explore the solution space effectively. Comparative evaluations on benchmark optimization problems indicate that CBKA consistently outperforms the standard BKA, confirming the positive impact of chaotic mapping on metaheuristic optimization performance [10]. Figure 4 presents the workflow of the Chaotic Black-Winged Kite Algorithm.

### 3.5. Description of the Proposed LBKA

**Figure 5:**
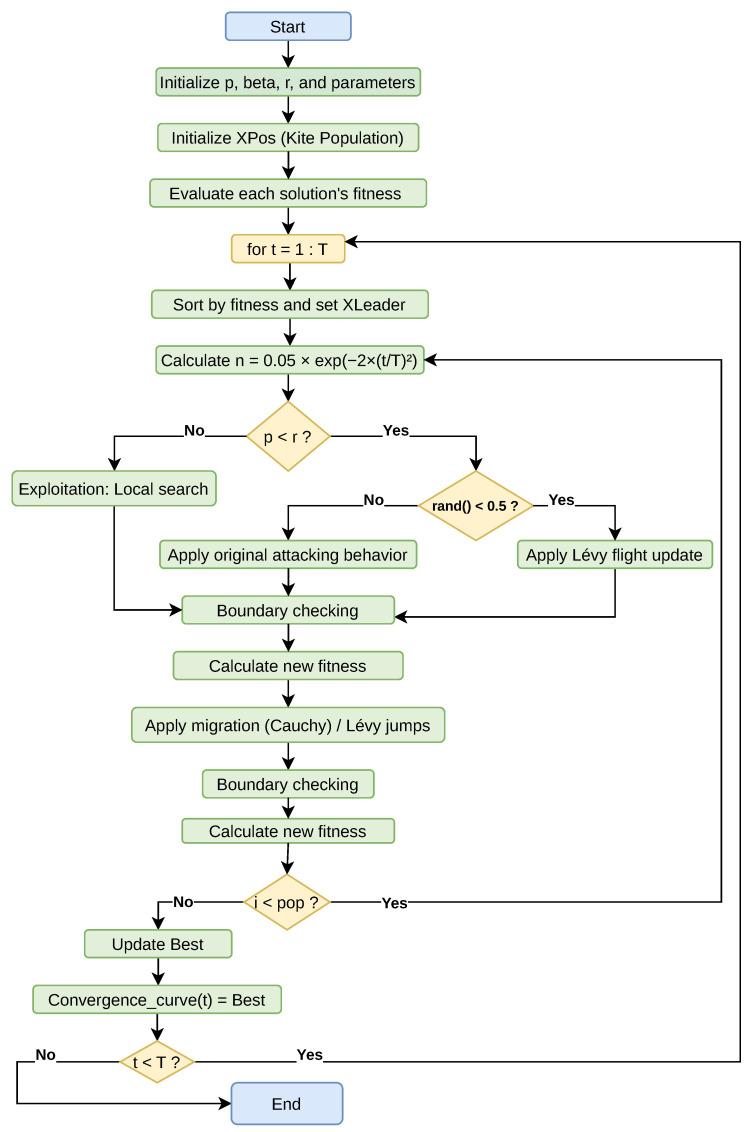
Workflow of the Lévy Black-Winged Kite Algorithm (LBKA).

The proposed Lévy Flight-based Black-Winged Kite Algorithm (LBKA) incorporates Lévy flight mechanisms into the BKA framework to reinforce global exploration during the optimization process. Lévy flight is characterized by step-length distributions that generate infrequent but significant long-distance movements interspersed with shorter steps, enabling the algorithm to traverse distant regions of the search space efficiently. This property enhances the algorithm’s ability to explore complex landscapes and reduces the risk of stagnation in locally optimal regions. Through the integration of Lévy flight dynamics, LBKA achieves a more effective trade-off between global exploration and local exploitation. The stochastic yet structured movement patterns introduced by Lévy flight guide the search toward unexplored and potentially promising areas while preventing excessive confinement around suboptimal solutions. In the clustering context, LBKA is adapted to minimize the Sum of Squared Errors (SSE) between data points and their closest centroids. Each individual encodes a set of *k* centroids in a *d*-dimensional space. At each iteration, data samples are assigned to the nearest centroid using Euclidean distance, and centroids are updated via Lévy flight-driven steps. This allows the algorithm to explore diverse clustering configurations while improving convergence stability. As a result, LBKA demonstrates improved convergence behavior, higher solution accuracy, and increased robustness compared to the standard BKA across various benchmark optimization problems. The LBKA procedure consists of population initialization, fitness evaluation, leader selection, adaptive parameter adjustment, Lévy flight-based position updates, boundary handling, fitness comparison, and diversity-preserving migration, with these steps iteratively executed until the predefined stopping criterion is satisfied [10]. The workflow of the Lévy Black-Winged Kite Algorithm (LBKA) is illustrated in Figure 5.

### 3.6. Description of the Proposed CLBKA

The proposed Chaotic Lévy-based Black-Winged Kite Algorithm (CLBKA) integrates Lévy flight and logistic chaotic mapping into the BKA framework to jointly enhance global exploration and local exploitation. Lévy flight enables occasional long-distance moves that facilitate escape from local optima, while the logistic chaotic map adaptively regulates control parameters through non-periodic dynamics, promoting diverse and non-repetitive search trajectories. Together, these mechanisms establish a synergistic search strategy in which Lévy flight supports wide-range exploration and chaotic control guides convergence. In addition, Cauchy-distributed perturbations applied during the migration phase further improve population diversity and mitigate premature convergence. Consequently, CLBKA demonstrates superior performance compared to the standard BKA and its single-enhanced variants, CBKA and LBKA [10]. While chaotic maps and Lévy flight have individually been employed in prior metaheuristics, CLBKA introduces a distinct hybrid integration strategy that tightly couples dynamic parameter control with conditionally triggered exploration. Unlike conventional approaches where chaotic maps are restricted to population initialization or random number generation, logistic chaos in CLBKA is applied at every iteration to modulate both attack and migration behaviors. Lévy flight is selectively activated with a fixed probability during the attack phase and conditionally re-invoked during migration when stagnation is detected relative to the population mean, thereby preserving long-range search capability without inducing excessive randomness. Together with Cauchy-based diversity enhancement, these components form a non-trivial extension of the BKA framework tailored for centroid-based clustering.

In CLBKA, each individual represents a set of *k* cluster centroids in a *d*-dimensional space. The algorithm is adapted for clustering by minimizing the Sum of Squared Errors (SSE) between data points and their nearest centroids. At each iteration, data points are assigned to the closest centroid using Euclidean distance, and centroid positions are updated to reduce intra-cluster distances. This formulation transforms clustering into a continuous optimization task, enabling CLBKA to efficiently search for optimal clustering configurations.

To clarify the joint integration of chaotic mapping, Lévy flight, and Cauchy mutation, their phase-specific roles within the optimization process are summarized as follows:

• Chaotic Logistic Maps are used at each iteration to modulate control parameters such as r, which affect both attack and migration behaviors. Their deterministic yet sensitive nature ensures dynamic variation, preventing stagnation and cyclic search patterns.
• Lévy Flight is selectively employed during the attack phase with a fixed probability and conditionally re-triggered during migration under stagnation, facilitating escape from local optima through long-range exploration.
• Cauchy Mutation operates in the migration phase, introducing localized high-kurtosis perturbations that enhance population diversity while maintaining convergence stability.

These mechanisms are mathematically complementary: chaos provides adaptive non-repetitive control, Lévy flight supports probabilistic global exploration, and Cauchy mutation ensures localized stochastic diversity. Their coordinated integration yields a tiered stochastic architecture that balances exploration, exploitation, and diversity, resulting in improved convergence stability and superior clustering performance across diverse datasets. The pseudocode of the proposed CLBKA is provided in Algorithm 2.
**Algorithm 2.** Pseudo-code of CLBKA [10].Algorithm: BKA with Lévy Flight and Chaotic Map 
**Initialize positions X and evaluate fitness****Set Lévy parameter β and chaos parameter r**

  3.**For t = 1 to T do**      -**Update r using chaotic map**      -For each kite •Compute noise n•**If** p < r: **If r and** < **0.5:**  Apply Lévy flight**Else:**  $X_{i}$ = $X_{i}$ + n×(1 + sin(r))× $X_{i}$**Else:**  $X_{i}$ = $X_{i}$+ n×(2×rand − 1) + 1)× $X_{i}$•**Apply bounds and update if better**

          ●**Migration:** If mod (t, 20) == 0 and $X_{i}$ bad:  **Apply Lévy jump** **Else:**  **Use Cauchy noise for movement**

          ●**Apply bounds and update if better**
        -Update global best

  4.**Return best position and fitness**

**Figure 6:**
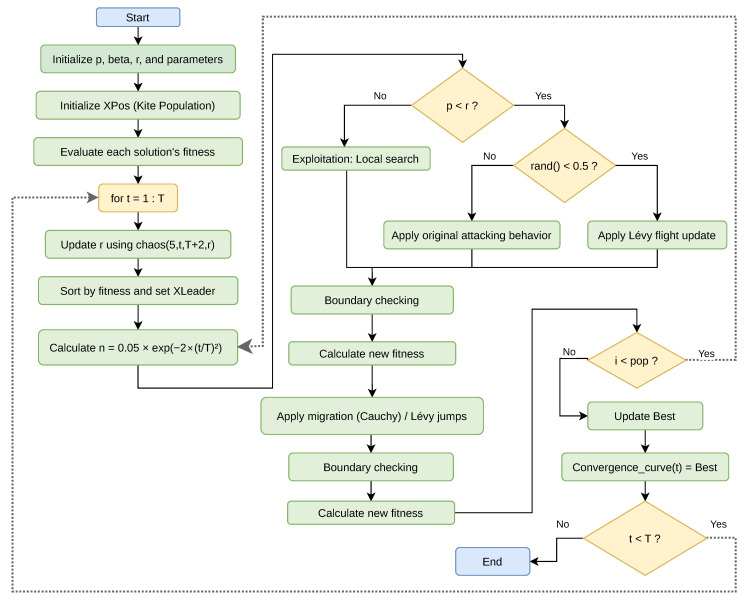
Schematic representation of the proposed CLBKA’s workflow.

Figure 6 presents the flowchart of the proposed Lévy-based Chaotic Black-Winged Kite Algorithm (CLBKA), outlining its main stages. The diagram depicts the overall algorithmic structure, covering parameter initialization, population creation, fitness assessment, chaotic updates, migration and attack phases, Lévy flight application, leader updating, and the iterative optimization procedure.

### 3.7. Clustering Problem

Clustering methods aim to divide datasets with unknown class labels into meaningful subgroups by grouping data samples that share similar characteristics [42]. The fundamental objective of clustering is to form clusters in which data points within the same group exhibit high similarity, while data points belonging to different groups are well separated. Accordingly, an effective clustering solution seeks to minimize intra-cluster distances while simultaneously maximizing inter-cluster separation [60,61].

Clustering is typically applied in scenarios where prior information about the underlying structure of the dataset is unavailable. Given a set of n observations drawn from a population, each observation is treated as a data instance characterized by a set of variables. During the clustering process, data instances with comparable properties are assigned to the same cluster, enabling the aggregation of observations while preserving essential information content. This process facilitates the discovery of inherent patterns within the data with minimal information loss [62].

In this study, the clustering task is formulated as a centroid-based optimization problem, where the objective is to minimize the Sum of Squared Errors (SSE), a widely adopted criterion in unsupervised learning. The objective function is defined as:

(10) $$SSE=\sum_{i=1}^{k}\sum_{x_{j}\in C_{i}}∥x_{j}-\mu_{i}{∥}^{2}$$

where $C_{i}$ represents the $i^{th}\;$ cluster, $\mu_{i}$ is the centroid of cluster $C_{i}$, and $x_{j}$ is a data point assigned to that cluster. The norm ∥⋅∥ denotes the Euclidean distance, which was selected due to its simplicity, interpretability, and common usage in clustering literature. While squared distances are accumulated in the algorithm, the implementation also includes a square root operation at the final level to improve numerical stability in optimization. No feature standardization or normalization procedure was applied prior to the optimization process. The clustering algorithm operates directly on the original feature values of each dataset. Therefore, distance calculations reflect the inherent scale and distribution characteristics of the datasets used in the experiments.

### 3.8. Dataset

In this study, sixteen benchmark datasets for classification and clustering were selected from the UCI (University of California, Irvine) Machine Learning Repository [63]. These datasets were chosen to evaluate the performance of the proposed methods under varying conditions, including different numbers of features, cluster centers, and data instances. For each dataset, the number of clusters (k) was set equal to the number of distinct class labels, as commonly adopted in benchmarking studies using labeled UCI datasets. While this setting enables a direct comparison with ground-truth labels, it is acknowledged that in real-world unsupervised scenarios the true number of clusters is typically unknown. Estimating k therefore constitutes an important extension of clustering algorithms. Future research may explore incorporating automatic k estimation into the CLBKA framework, either through internal validation indices or by treating k as an optimization variable within the metaheuristic search process. Prior to clustering, a uniform preprocessing procedure was applied across all datasets. Missing values, when present, were handled using mean imputation based on the corresponding feature column. No additional normalization or standardization was applied, and all datasets were processed in their original numerical form. The same preprocessing protocol was consistently used for all datasets to ensure methodological fairness and reproducibility. An overview of the datasets and their key characteristics is provided in Table 1.

### 3.9. Friedman Test

The Friedman test is a non-parametric statistical method commonly employed to compare multiple related samples, especially when parametric assumptions such as normality are violated [64]. In optimization research, it is frequently used to assess the comparative performance of several algorithms over multiple benchmark problems by ranking their results. Based on these rankings, the test determines whether statistically significant performance differences exist among the algorithms. Owing to its suitability for dependent samples and its distribution-free nature, the Friedman test has become a standard tool for algorithm evaluation in benchmark-based studies [65]. To statistically validate the comparative performance of the algorithms across multiple datasets, the Friedman test was employed. The null hypothesis (H_0_) states that all algorithms perform equivalently and therefore have equal median ranks across the considered benchmark datasets. The alternative hypothesis (H_1_) states that at least one algorithm performs significantly differently. For each dataset, the algorithms were ranked according to their performance, measured in terms of Sum of Squared Errors (SSE) and Rand Index (RI), where rank 1 was assigned to the best-performing algorithm and higher ranks to inferior ones. In cases of ties, average ranks were assigned. The mean rank of each algorithm across all datasets was then computed and used in the Friedman test statistic. The significance level was set to α = 0.05. If the Friedman test indicated statistically significant differences, post hoc pairwise comparisons were conducted using the Nemenyi test to identify which algorithms differed significantly.

The Friedman test was preferred over parametric alternatives because the distributional assumptions of normality and homoscedasticity cannot be guaranteed for performance metrics across heterogeneous benchmark datasets. Compared to other non-parametric alternatives such as the Quade test, the Friedman test is widely adopted in multi-dataset algorithm comparison studies due to its robustness and interpretability.

### 3.10. Wilcoxon Signed-Rank Test

The Wilcoxon signed-rank test is a well-established non-parametric method used to assess differences between two dependent samples, such as paired observations or repeated measurements taken from the same population. In contrast to the paired t-test, this approach does not rely on the assumption of normally distributed data, which makes it particularly appropriate when the underlying distribution is unknown or deviates from normality. For this reason, it is frequently employed in optimization and metaheuristic algorithm studies to perform pairwise comparisons of algorithmic performance across multiple benchmark functions, where outcomes are typically obtained from several independent executions [66,67].

### 3.11. Time Complexity Analysis

This section presents a theoretical analysis of the computational complexity of the proposed Black-Winged Kite Algorithm (BKA) and its enhanced variants (CBKA, LBKA, and CLBKA), in order to substantiate the claims regarding computational efficiency. Let the following notations be defined:

• n: Population size (number of candidate solutions);
• d: Dimensionality of the dataset (number of features);
• k: Number of clusters;
• T: Maximum number of iterations.

In centroid-based metaheuristic clustering frameworks, the dominant computational cost arises from the fitness evaluation process, which requires assigning data samples to cluster centroids and computing the clustering objective function, typically the sum of squared errors (SSE). For each candidate solution, the assignment of data points to the nearest cluster centroid involves distance calculations with a time complexity proportional to O(k⋅d). Consequently, the evaluation of the objective function for a single candidate solution also scales as O(k⋅d). During each iteration, every search agent undergoes a sequence of operations including position updates (attack and migration behaviors), fitness evaluation, and leader comparison. Therefore, the overall computational cost per iteration across the entire population can be expressed as:

O(n⋅k⋅d)

Over T iterations, the total time complexity of the algorithm becomes:

O(n⋅k⋅d⋅T)

The proposed enhancements introduced in CBKA, LBKA, and CLBKA, namely chaotic logistic mapping and Lévy flight mechanisms, primarily consist of stochastic perturbations, random number generation, and elementary mathematical operations. These operations are executed in constant time and do not introduce additional nested loops or population-level evaluations. As a result, they do not alter the asymptotic order of the computational complexity. Accordingly, despite incorporating additional exploration mechanisms, the CLBKA preserves the same theoretical time complexity as the standard BKA. The improvements in clustering performance achieved by CLBKA are therefore obtained without increasing the algorithm’s asymptotic computational burden, indicating a favorable trade-off between solution quality and computational cost.

## 4. Result and Discussion

### 4.1. Comparative Analysis of BKA, CBKA, LBKA, and CLBKA

In this study, the clustering performance of four BKA-based approaches (BKA, LBKA, CBKA, and CLBKA) was compared under different combinations of population size (P = 30, P = 50) and iteration number (T = 500, T = 1000). An examination of the average objective function values and rankings reported in the tables shows that CLBKA achieves the best or an equally good performance across all datasets. This finding indicates that CLBKA can establish a more effective balance between exploration and exploitation during the search process, thereby reducing the likelihood of being trapped in local minimum. In contrast, the classical BKA tends to remain at higher objective function values for most datasets, while LBKA and CBKA provide a noticeable improvement over BKA but still lag behind CLBKA.

This superiority of CLBKA is most evident under limited resource settings (P = 30, T = 500), where both computational budget and population diversity are constrained. The integration of Lévy flight and chaotic logistic mapping equips CLBKA with two complementary mechanisms: Lévy-based jumps allow the algorithm to break free from local optima through long-distance exploration, while chaos-driven updates introduce controlled diversity, preventing premature convergence. These characteristics are especially critical in complex datasets like Glass and Btisuse, where high-dimensional features and class overlap commonly hinder traditional metaheuristics. Indeed, CLBKA not only yields the lowest average SSE in these datasets but also maintains significantly lower standard deviations, confirming its stability across runs. Moreover, the performance gap between CBKA/LBKA and CLBKA highlights the nonlinear synergy achieved by combining chaos and Lévy dynamics within a single framework. CBKA’s performance is enhanced by diversity but suffers from limited reach, while LBKA explores widely but lacks adaptive control. CLBKA successfully unifies these strengths, allowing it to both explore the global search space and converge efficiently when promising regions are identified. The results from Diabetes and Parkinson datasets, where overlapping clusters pose difficulty, further support this, as CLBKA remains robust where other algorithms degrade. 

Overall, CLBKA’s performance is not only numerically superior but also structurally justified, demonstrating that thoughtful hybridization of metaheuristic strategies can yield both accuracy and consistency, especially in low-budget clustering scenarios. 

To ensure reproducibility and enable robust statistical evaluation, all algorithms were independently executed 30 times on each dataset. For each algorithm–dataset pair, the best, worst, mean, and standard deviation values of the clustering objective function were computed. The hyperparameter settings used in all experiments are provided in Table 2, and the results are summarized in Table 3, Table 4, Table 5, Table 6.

**Table 2:** Hyperparameter Settings Used in the Experiments of proposed methods.

Parameter	Value(s) Used
Population Size (P)	30, 50
Iteration Number (T)	500, 1000
Independent Runs	30
Lévy Flight Parameter (β)	1.5
Logistic Map Type	Logistic Map
Initial Chaos Value (x_0_)	0.7
Probability Parameter (p)	0.9
Migration Coefficient (m)	$2\times sin(r+\frac{\pi }{2})$

**Table 3:** Comparative Clustering Results: BKA, LBKA, CBKA, and CLBKA (P = 30, T = 500).

P = 30, T = 500					
Dataset		BKA	LBKA	CBKA	CLBKA
**Balance**	B	1434.10	1423.86	1423.85	**1423.84**
	W	1456.28	1423.96	1423.97	**1423.88**
	A	1447.66	1423.89	1423.88	**1423.87**
	S	5.77632	0.02325	0.02367	**0.01189**
	Rank	4	3	2	**1**
**Credit**	B	563695	556747	556753	**556746**
	W	606112	557209	557211	**557159**
	A	583138	556953	556982	**556834**
	S	11354.3	197.967	214.150	**130.690**
	Rank	4	2	3	**1**
**Dermatology**	B	3088.18	2812.45	2793.26	**2773.17**
	W	3243.33	2912.23	2934.34	**2860.30**
	A	3182.72	2861.85	2860.77	**2838.48**
	S	33.0327	26.9815	31.8901	**22.9783**
	Rank	4	3	2	**1**
**E. coli**	B	104.354	68.9967	68.5131	**69.8495**
	W	123.874	74.7430	74.8069	**73.9756**
	A	115.568	72.2632	72.5915	**72.1794**
	S	5.30365	1.76415	1.60913	**1.44045**
	Rank	4	2	3	**1**
**Glass**	B	418.437	294.017	283.512	**281.92**
	W	491.441	332.000	326.243	**308.887**
	A	457.13	308.743	305.671	**299.149**
	S	19.528	9.03923	11.5815	**6.8524**
	Rank	4	3	2	**1**
**Iris**	B	129.504	96.694	96.7011	**96.7043**
	W	170.6	97.164	97.5998	**96.8414**
	A	148.19	96.8274	96.8728	**96.7764**
	S	8.9912	0.116003	0.226534	**0.042248**
	Rank	4	2	3	**1**
**Thyroid**	B	2493.49	1874.54	1878.42	**1875.11**
	W	2878.4	1967.05	2000.6	**1922.1**
	A	2691.15	1912.56	1908.09	**1899.24**
	S	105.013	26.6018	23.8593	**12.5892**
	Rank	4	3	2	**1**
**Wine**	B	16484	16315.3	16310.6	**16316**
	W	17214.8	16343.6	16344.5	**16329.7**
	A	16811.5	16326	16326.2	**16324.5**
	S	184.079	6.36058	7.39038	**3.86568**
	Rank	4	2	3	**1**
**Heart**	B	9819.98	9442.35	9442.45	**9441.81**
	W	10791.4	9446.31	9445.42	**9443.94**
	A	10314.4	9443.84	9443.74	**9443.25**
	S	263.779	0.894661	0.739588	**0.466652**
	Rank	4	3	2	**1**
**Spect**	B	593.746	555.296	554.817	**555.056**
	W	636.271	561.334	561.966	**557.343**
	A	620.493	557.417	558.056	**556.296**
	S	11.5109	1.3916	1.61934	**0.70751**
	Rank	4	2	3	**1**
**Diabets**	B	73433.6	72107.2	72107.2	**72107.2**
	W	81335.4	72186.1	74100.5	**72107.3**
	A	75681.3	72109.9	72173.7	**72107.2**
	S	1879.75	14.4001	363.91	**0.01464**
	Rank	4	2	3	**1**
**Hepatit**	B	9792.76	9442.77	9442.2	**9441.99**
	W	11014.9	9446.02	9445.64	**9443.95**
	A	10384.3	9443.82	9443.8	**9443.31**
	S	314.844	0.79544	0.76853	**0.46028**
	Rank	4	3	2	**1**
**Btissue**	B	198575	130817	128806	**129383**
	W	274666	151215	152176	**140714**
	A	239229	140130	137016	**135735**
	S	17800.9	5799.35	5802.75	**3439.14**
	Rank	4	3	2	**1**
**Parkinson**	B	16466.5	**16463**	**16463**	**16462.9**
	W	16732.8	**16463.1**	**16463.2**	**16463**
	A	16547.5	**16463**	**16463**	**16463**
	S	65.1892	**0.04143**	**0.04846**	**0.02922**
	Rank	2	**1**	**1**	**1**
**Somerville**	B	302.765	280.534	280.529	**280.528**
	W	327.903	280.642	280.669	**280.569**
	A	318.476	280.567	280.579	**280.553**
	S	5.72583	0.02164	0.03257	**0.01052**
	Rank	4	2	3	**1**
**User Modeling**	B	108.83	97.4787	97.581	**97.4115**
	W	118.593	99.3549	100.63	**98.9282**
	A	113.159	98.2043	99.0395	**98.1158**
	S	2.35814	0.44138	0.93239	**0.48966**
	Rank	4	2	3	**1**

As shown in Table 4, increasing the number of iterations to P = 50 while keeping T = 1000 improves the clustering performance of all methods; however, this improvement is markedly less pronounced for CLBKA. This indicates that CLBKA is able to reach high-quality solutions at earlier stages of the search, whereas BKA, LBKA, and CBKA require extended iterations to compensate for slower convergence. The early saturation behavior of CLBKA can be attributed to the combined effect of chaotic parameter control and Lévy flight exploration, which enables efficient global search in the early iterations while rapidly refining promising regions. The reduced performance gap at higher iteration budgets suggests that prolonged search primarily benefits algorithms lacking strong diversification mechanisms. In contrast, CLBKA maintains both low SSE values and low variance across datasets with different structural characteristics, such as Glass and User Modeling, indicating stable convergence behavior. These results confirm that the hybrid design of CLBKA not only enhances exploration but also reduces sensitivity to iteration count, making it effective under both limited and extended computational budgets.

**Table 4:** Comparative Clustering Results: BKA, LBKA, CBKA, and CLBKA (P = 30, T = 1000).

P = 30, T = 1000					
Dataset		BKA	LBKA	CBKA	CLBKA
**Balanc** **e**	B	1434.38	1423.84	1423.84	**1423.84**
	W	1454.41	1423.88	1423.88	**1423.86**
	A	1443.92	1423.86	1423.85	**1423.84**
	S	4.45921	0.01023	0.00822	**0.00544**
	Rank	4	3	2	**1**
**Credit**	B	566003	556740	556747	**556742**
	W	599243	557158	594453	**556977**
	A	579439	556835	558203	**556781**
	S	9678.94	149.21	6849.51	**56.1696**
	Rank	4	2	3	**1**
**Dermatology**	B	3059.21	2660.7	2638.85	**2625.25**
	W	3239.09	2733.97	2746.20	**2704.36**
	A	3158.23	2694.52	2703.09	**2671.65**
	S	36.1588	18.6159	21.5782	**20.2732**
	Rank	4	2	3	**1**
**E. coli**	B	101.197	68.609	65.9677	**67.2796**
	W	122.595	73.1475	72.9009	**70.6152**
	A	112.162	70.1578	70.2365	**69.6348**
	S	5.06733	1.10130	1.48482	**0.69459**
	Rank	4	2	3	**1**
**Glass**	B	366.561	275.199	270.708	**276.05**
	W	481.605	309.463	322.238	**290.102**
	A	440.211	289.142	293.247	**283.884**
	S	26.6856	9.33814	13.1209	**4.05847**
	Rank	4	2	3	**1**
**Iris**	B	120.712	96.6762	96.6927	**96.6689**
	W	158.881	96.6762	96.7691	**96.7143**
	A	143.953	96.7076	96.7177	**96.6996**
	S	7.54585	0.01884	0.01996	**0.00935**
	Rank	4	2	3	**1**
**Thyroid**	B	2197.45	1868.05	1868.88	**1868.35**
	W	2864.43	1902.06	1912.14	**1890.24**
	A	2625.51	1881.37	1882.25	**1873.42**
	S	127.693	11.036	13.6823	**4.44119**
	Rank	4	2	3	**1**
**Wine**	B	16533.7	16311.3	16309.9	**16307.8**
	W	16971.8	16336.4	16328.4	**16318.7**
	A	16705.4	16318.1	16316.8	**16314.6**
	S	104.974	6.3339	5.20692	**3.02425**
	Rank	4	3	2	**1**
**Heart**	B	9765.08	9441.12	9441.36	**9441.24**
	W	10898.4	9443.55	9443.79	**9442.23**
	A	10306.5	9442.18	9442.22	**9441.82**
	S	307.755	0.51988	0.55174	**0.23585**
	Rank	4	2	3	**1**
**Spect**	B	601.502	554.546	554.546	**554.546**
	W	632.728	554.553	554.565	**554.548**
	A	612.34	554.549	554.549	**554.547**
	S	7.55607	0.00160	0.003451	**0.000612**
	Rank	3	2	2	**1**
**Diabets**	B	72696.8	72107.2	72107.2	**72107.2**
	W	79053.1	72107.2	72186.1	**72107.2**
	A	74839.6	72107.2	72109.9	**72107.2**
	S	1536	0.000428	14.4065	**5.05836 × 10^−5^**
	Rank	3	**1**	2	**1**
**Hepatit**	B	9822.05	9441.4	9441.6	**9440.88**
	W	10823.9	9442.98	9443.43	**9442.23**
	A	10204.5	9442.18	9442.29	**9441.85**
	S	251.655	0.41334	0.46952	**0.32672**
	Rank	4	2	3	**1**
**Btissue**	B	197898	127761	126541	**127535**
	W	263980	140316	149688	**132089**
	A	228515	131256	132632	**129099**
	S	14765.6	3028.01	5987.26	**1253.76**
	Rank	4	2	3	**1**
**Parkinson**	B	16480.7	**16462.9**	**16462.9**	**16462.9**
	W	16693.3	**16463.1**	**16463.1**	**16463**
	A	16530.4	**16463**	**16463**	**16463**
	S	45.3018	**0.0469**	**0.0495**	**0.0232**
	Rank	2	**1**	**1**	**1**
**Somerville**	B	290.487	280.526	280.52	**280.516**
	W	325.498	280.555	280.574	**280.537**
	A	313.688	280.537	280.542	**280.53**
	S	8.51044	0.008492	0.011889	**0.00506**
	Rank	4	2	3	**1**
**User Modeling**	B	106.622	97.3557	97.3582	**97.3519**
	W	115.721	97.5257	98.3507	**97.3872**
	A	111.921	97.3761	97.4746	**97.3697**
	S	2.13493	0.030232	0.23340	**0.00866**
	Rank	4	2	3	**1**

As seen in Table 5, increasing the population size to P = 50 while keeping T = 500 improves the performance of all methods due to greater solution diversity and wider search coverage. However, CLBKA maintains its leading position, delivering the lowest average SSE values and smallest standard deviations across most datasets. This indicates that CLBKA’s hybrid design enables it to leverage a smaller population more effectively, making additional population size less critical for its convergence behavior. In contrast, BKA, LBKA, and CBKA benefit more from population growth, as they rely on a larger swarm to escape local minima and enhance search stability. Yet even with this advantage, they fall short of CLBKA’s results, highlighting that the integration of chaotic perturbations and Lévy-based jumps provides a more powerful mechanism for maintaining exploration without relying solely on population size. Notably, CLBKA performs best even in challenging datasets like Btissue and Glass, where high dimensionality and complex cluster structures typically degrade algorithm performance.

**Table 5:** Comparative Clustering Results: BKA, LBKA, CBKA, and CLBKA (P = 50, T = 500).

P = 50, T = 500					
Dataset		BKA	LBKA	CBKA	CLBKA
**Balance**	B	1436.41	1423.84	1423.85	**1423.84**
	W	1454.58	1423.91	1423.91	**1423.88**
	A	1444.36	1423.88	1423.88	**1423.86**
	S	4.7041	0.0157778	0.0135112	**0.00911**
	Rank	3	2	2	**1**
**Credit**	B	565283	556745	556748	**556745**
	W	597568	557209	557210	**556806**
	A	581213	556945	556979	**556778**
	S	9879.75	198.885	204.014	**25.6746**
	Rank	4	2	3	**1**
**Dermatology**	B	3027.8	2769.86	2790.57	**2759.27**
	W	3210.58	2905.45	2900.54	**2856.32**
	A	3157.56	2852.73	2851	**2829.63**
	S	43.4983	28.4162	25.1494	**25.4440**
	Rank	4	3	2	**1**
**E. coli**	B	100.886	68.2585	69.2266	**68.6975**
	W	117.858	74.2742	74.8621	**72.6633**
	A	111.807	71.8115	72.1771	**71.2404**
	S	5.03679	1.51358	1.68655	**0.961163**
	Rank	4	2	3	**1**
**Glass**	B	358.529	275.855	280.364	**271.63**
	W	502.566	327.764	340.983	**298.615**
	A	436.177	301.786	301.395	**290.056**
	S	35.6932	12.7288	12.3373	**6.50043**
	Rank	4	3	2	**1**
**Iris**	B	125.972	96.7071	96.7029	**96.7166**
	W	164.667	96.87	96.96	**96.7779**
	A	143.97	96.7676	96.7794	**96.7458**
	S	8.37963	0.03711	0.06418	**0.01758**
	Rank	4	2	3	**1**
**Thyroid**	B	2450.23	1871.36	1874.99	**1871.41**
	W	2931.19	1931.84	1956.71	**1891.98**
	A	2637.05	1895.34	1901.33	**1880**
	S	129.63	17.9185	23.0753	**4.4781**
	Rank	4	2	3	**1**
**Wine**	B	16532.7	16315.9	16305.7	**16310.3**
	W	17288.9	16341.9	16344.7	**16328.2**
	A	16758.3	16326.3	16324.8	**16321.5**
	S	184.535	6.52263	7.85019	**4.38218**
	Rank	4	3	2	**1**
**Heart**	B	9833.54	9441.8	9441.9	**9441.91**
	W	10952.1	9445.4	9444.22	**9443.31**
	A	10218.6	9443.23	9443.06	**9442.72**
	S	258.891	0.74242	0.52740	**0.37627**
	Rank	4	3	2	**1**
**Spect**	B	588.584	555.479	555.047	**554.921**
	W	641.295	559.913	559.849	**557.19**
	A	616.065	557.228	556.872	**556.293**
	S	10.55	1.09841	1.16431	**0.71150**
	Rank	4	3	2	**1**
**Diabets**	B	73044.3	72107.2	72107.2	**72107.2**
	W	78234.3	72107.3	72107.3	**72107.2**
	A	75028	72107.2	72107.2	**72107.2**
	S	1338.63	0.01689	0.01421	**0.00451**
	Rank	2	**1**	**1**	**1**
**Hepatit**	B	9680.26	9441.86	9442.35	**9442.3**
	W	10815.6	9444.76	9444.61	**9443.2**
	A	10227.6	9443.18	9443.41	**9442.81**
	S	275.863	0.67621	0.61209	**0.26600**
	Rank	4	2	3	**1**
**Btissue**	B	207483	130477	129814	**129538**
	W	261335	146255	150189	**136673**
	A	230022	136942	134851	**132930**
	S	14587.6	4049.21	4628.85	**1856.65**
	Rank	4	3	2	**1**
**Parkinson**	B	16475.1	**16463**	**16462.9**	**16462.9**
	W	16652.7	**16463.1**	**16463**	**16463**
	A	16551.8	**16463**	**16463**	**16463**
	S	42.1915	**0.02986**	**0.03141**	**0.01874**
	Rank	2	**1**	**1**	**1**
**Somerville**	B	306.112	280.534	280.537	**280.525**
	W	331.67	280.58	280.882	**280.561**
	A	315.78	280.556	280.578	**280.545**
	S	6.83961	0.01063	0.06132	**0.00959**
	Rank	4	2	3	**1**
**User Modeling**	B	108.741	97.4006	97.3914	**97.4178**
	W	115.514	99.0021	100.41	**98.5497**
	A	111.938	98.07	98.4547	**97.9132**
	S	1.65219	0.51040	0.77553	**0.35806**
	Rank	4	2	3	**1**

Table 6 shows that increasing both the population size and the number of iterations leads to general performance gains across all methods. However, CLBKA consistently outperforms the others by achieving the lowest objective function values and standard deviations on nearly all datasets. This result confirms that its hybrid structure is effective not only in complex datasets like Btissue and Parkinson, but also in structured datasets such as Iris and Wine, and in overlapping-class datasets like Credit and Dermatology. The method’s ability to maintain high accuracy and stability under varying data characteristics highlights its robustness and adaptability, regardless of problem complexity.

**Table 6:** Comparative Clustering Results: BKA, LBKA, CBKA, and CLBKA (P = 50, T = 1000).

P = 50, T = 1000					
Dataset		BKA	LBKA	CBKA	CLBKA
**Balance**	B	1436.04	**1423.83**	**1423.84**	**1423.83**
	W	1449.26	**1423.86**	**1423.86**	**1423.85**
	A	1441.67	**1423.85**	**1423.85**	**1423.85**
	S	3.60507	**0.00631**	**0.00550**	**0.00411**
	Rank	2	**1**	**1**	**1**
**Credit**	B	564389	556740	556742	**556742**
	W	595991	557156	557210	**556802**
	A	573943	556788	556897	**556764**
	S	6897.1	101.561	195.646	**24.2983**
	Rank	4	2	3	**1**
**Dermatology**	B	3060.72	2635.59	2634.06	**2631.57**
	W	3201.69	2711.24	2742.23	**2691.70**
	A	3144.38	2682.16	2687.34	**2675.54**
	S	32.1685	17.19	27.3887	**15.4241**
	Rank	4	2	3	**1**
**E. coli**	B	102.277	**65.8747**	65.7057	67.9426
	W	117.586	**71.9892**	72.9776	70.1752
	A	110.625	**69.2800**	70.0311	69.3270
	S	3.78799	**1.29255**	1.36244	0.67581
	Rank	4	**1**	3	2
**Glass**	B	392.891	267.009	271.683	**267.761**
	W	460.379	306.057	305.949	**285.459**
	A	429.551	283.274	283.976	**279.932**
	S	17.3801	8.72428	9.98877	**4.67789**
	Rank	4	2	3	**1**
**Iris**	B	111.691	96.6753	96.6712	**96.6783**
	W	154.024	96.7284	96.7322	**96.7016**
	A	137.735	96.7017	96.7035	**96.6920**
	S	9.24844	0.01326	0.01348	**0.00602**
	Rank	4	2	3	**1**
**Thyroid**	B	2147.9	1869.31	1868.4	**1868.06**
	W	2739.08	1901.6	1899.67	**1872.65**
	A	2577.73	1879.56	1877.15	**1870.12**
	S	146.457	11.6675	11.2886	**1.30922**
	Rank	4	3	2	**1**
**Wine**	B	16398.2	16306.9	16307.3	**16307.9**
	W	16973.7	16320.4	16326.3	**16317.1**
	A	16646.7	16314.2	16316	**16313.6**
	S	122.127	3.14338	4.3693	**2.90356**
	Rank	4	2	3	**1**
**Heart**	B	9677.21	9441.14	9441.13	**9441.12**
	W	10511	9442.12	9442.4	**9441.85**
	A	10034.5	9441.69	9441.83	**9441.61**
	S	207.995	0.26810	0.34925	**0.16645**
	Rank	4	2	3	**1**
**Spect**	B	594.06	554.546	554.546	**554.545**
	W	621.359	554.55	554.555	**554.548**
	A	609.532	554.548	554.548	**554.547**
	S	7.63409	0.00098	0.00164	**0.00064**
	Rank	3	2	2	**1**
**Diabets**	B	72639.5	72107.2	72107.2	**72107.2**
	W	75818	72107.2	72107.2	**72107.2**
	A	73965.8	72107.2	72107.2	**72107.2**
	S	783.249	0.000104	2.39579 × 10^−5^	**4.11171 × 10^−6^**
	Rank	2	**1**	**1**	**1**
**Hepatit**	B	9804.35	9441.46	9441.24	**9440.95**
	W	10750.8	9443.03	9442.92	**9441.87**
	A	10144.1	9441.92	9441.88	**9441.54**
	S	201.581	0.37796	0.45188	**0.22902**
	Rank	4	3	2	**1**
**Btissue**	B	196346	126316	126719	**126236**
	W	237662	143134	137434	**130039**
	A	218282	132009	129590	**128231**
	S	13020.3	4507.11	2474.58	**1129.44**
	Rank	4	3	2	**1**
**Parkinson**	B	16478.2	**16462.9**	**16462.9**	**16462.9**
	W	16574.1	**16463.1**	**16463.1**	**16463**
	A	16510	**16463**	**16463**	**16463**
	S	25.9548	**0.02985**	**0.04561**	**0.01986**
	Rank	2	**1**	**1**	**1**
**Somerville**	B	298.905	280.519	280.518	**280.522**
	W	322.133	280.552	280.555	**280.538**
	A	311.296	280.534	280.536	**280.532**
	S	5.23741	0.00741	0.00799	**0.00358**
	Rank	4	2	3	**1**
**User Modeling**	B	105.134	97.3535	97.3575	**97.3576**
	W	113.24	97.3950	98.5556	**97.3766**
	A	110.37	97.3653	97.4594	**97.3649**
	S	1.65405	0.00818	0.27228	**0.00475**
	Rank	4	2	3	**1**

**Figure 7:**
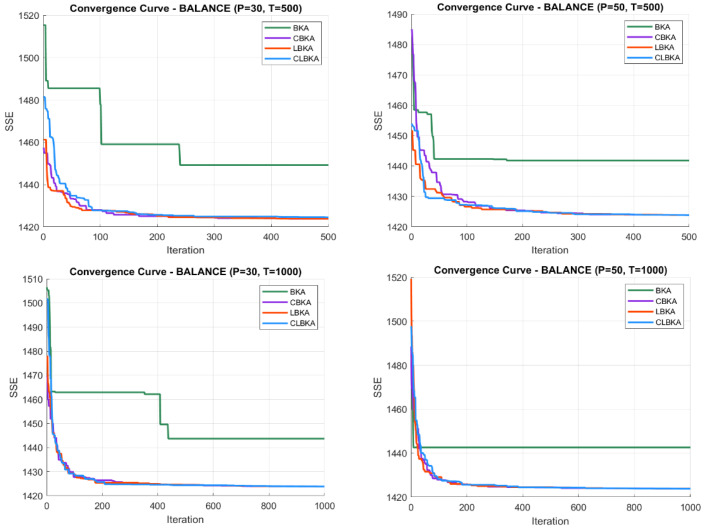
Convergence of the total square distance for **Balance** data set.

**Figure 8:**
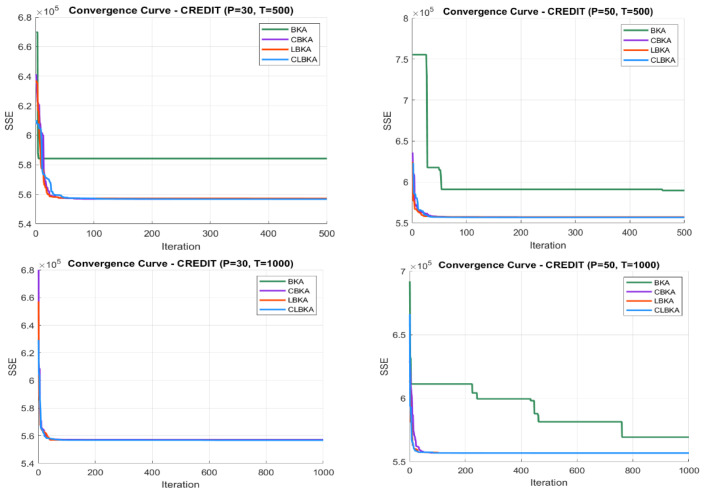
Convergence of the total square distance for **Credit** data set.

**Figure 9:**
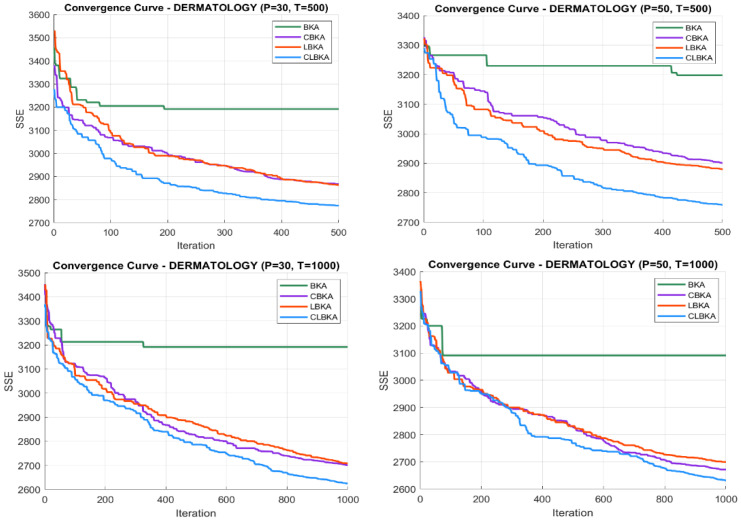
Convergence of the total square distance for **Dermatology** data set.

**Figure 10:**
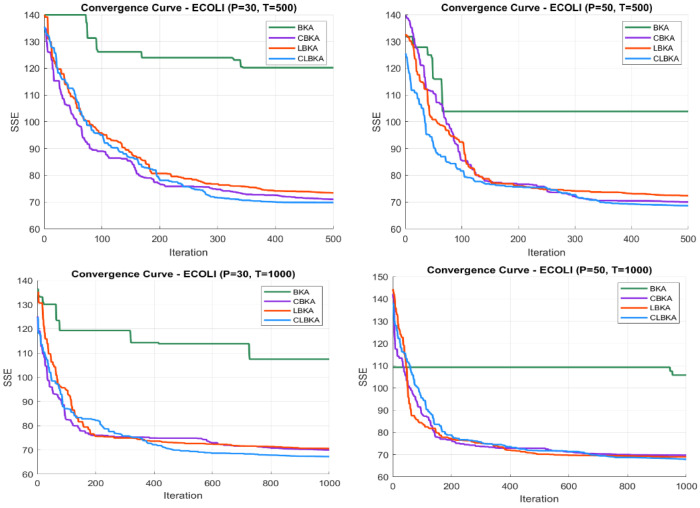
Convergence of the total square distance for **E. coli** data set.

**Figure 11:**
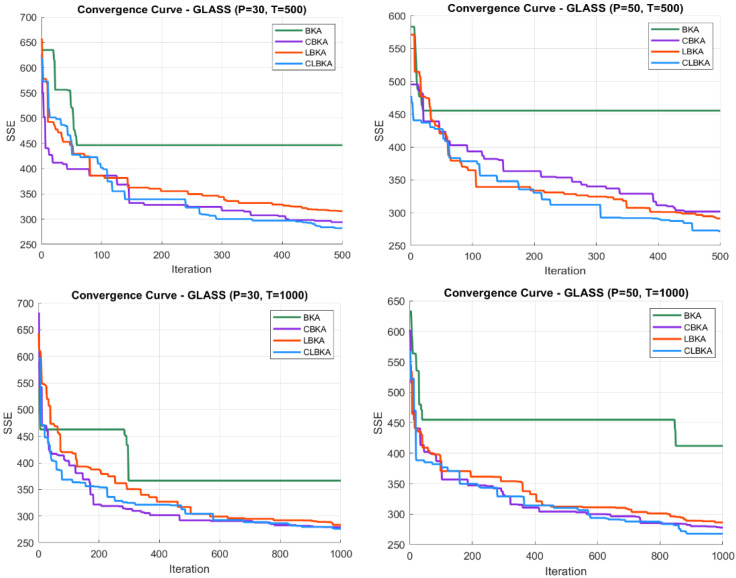
Convergence of the total square distance for **Glass** data set.

**Figure 12:**
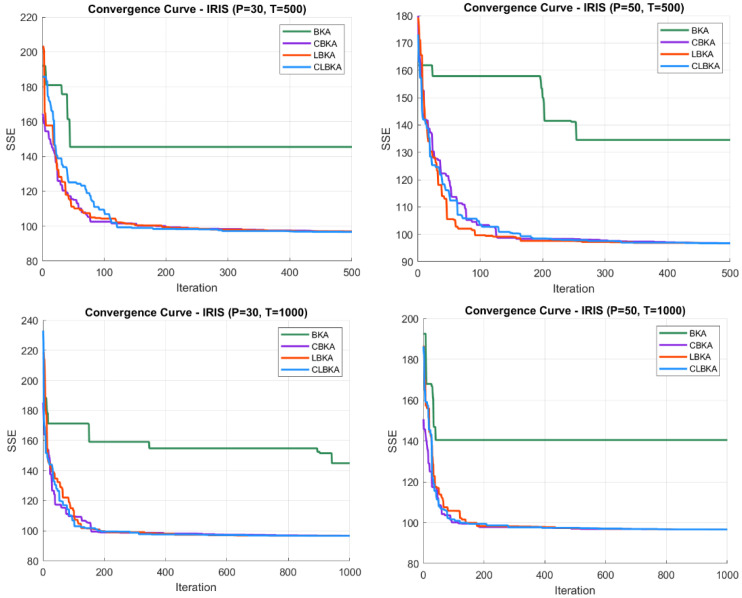
Convergence of the total square distance for **Iris** data set.

**Figure 13:**
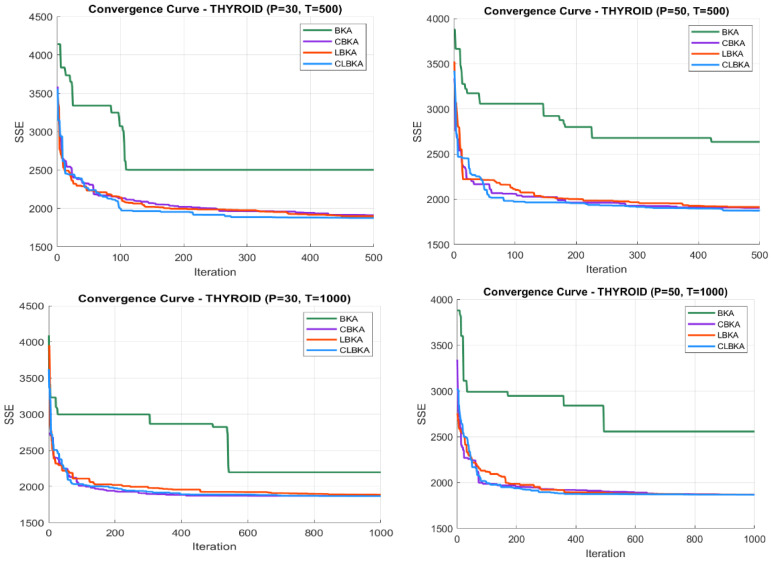
Convergence of the total square distance for **Thyroid** data set.

**Figure 14:**
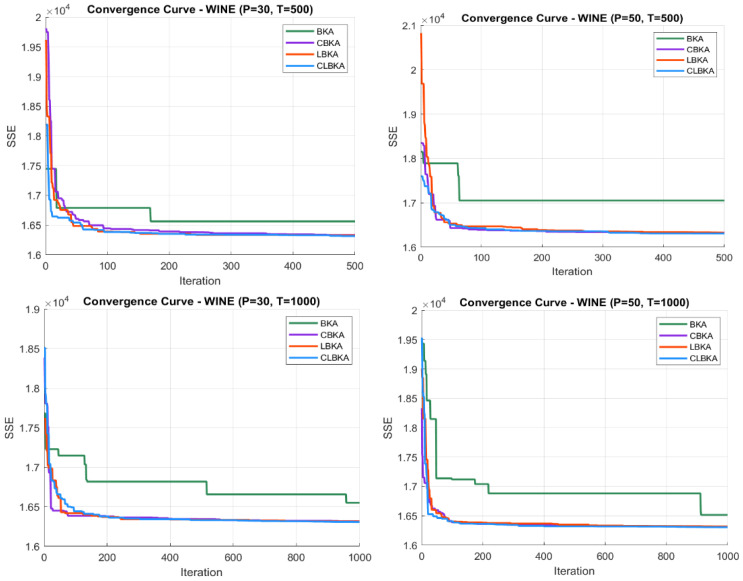
Convergence of the total square distance for **Wine** data set.

**Figure 15:**
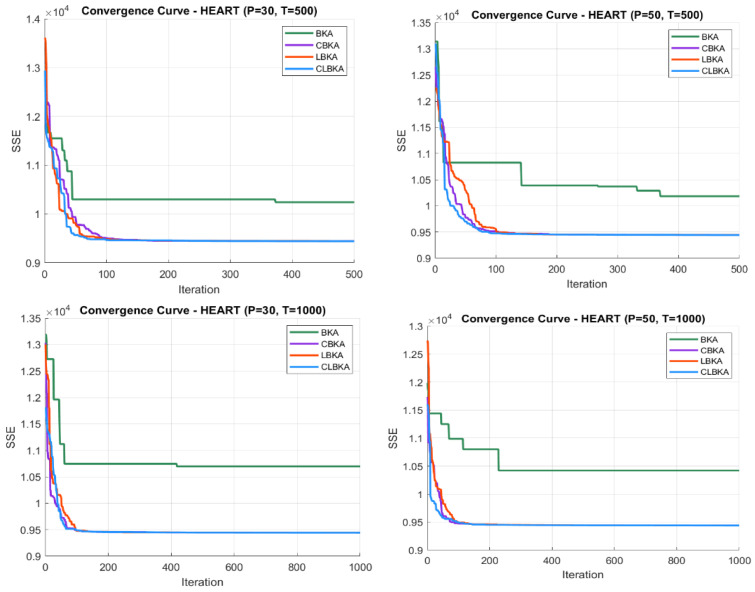
Convergence of the total square distance for **Heart** data set.

**Figure 16:**
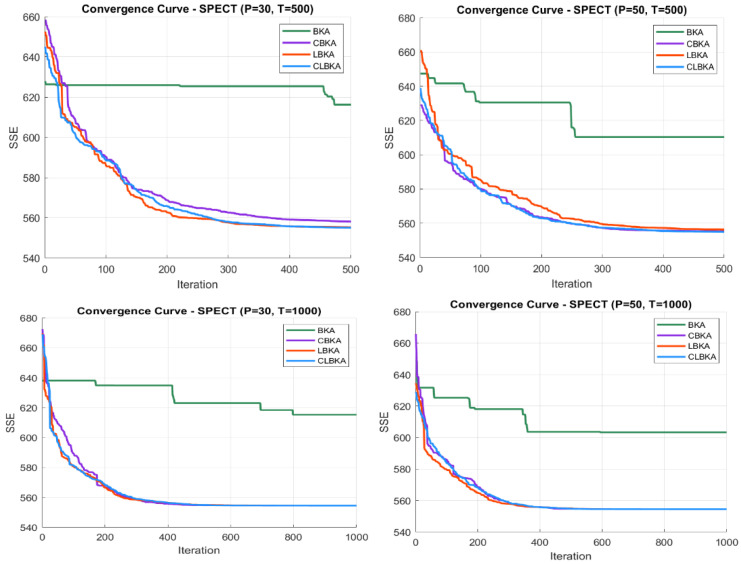
Convergence of the total square distance for **Spect** data set.

**Figure 17:**
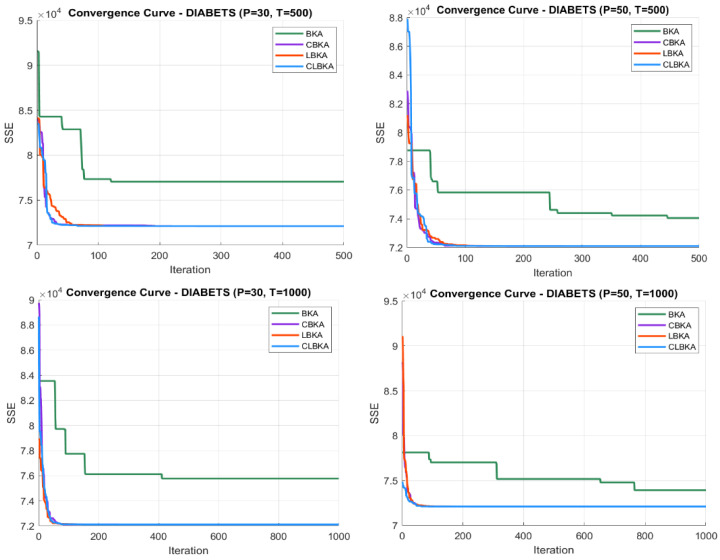
Convergence of the total square distance for **Diabets** data set.

**Figure 18:**
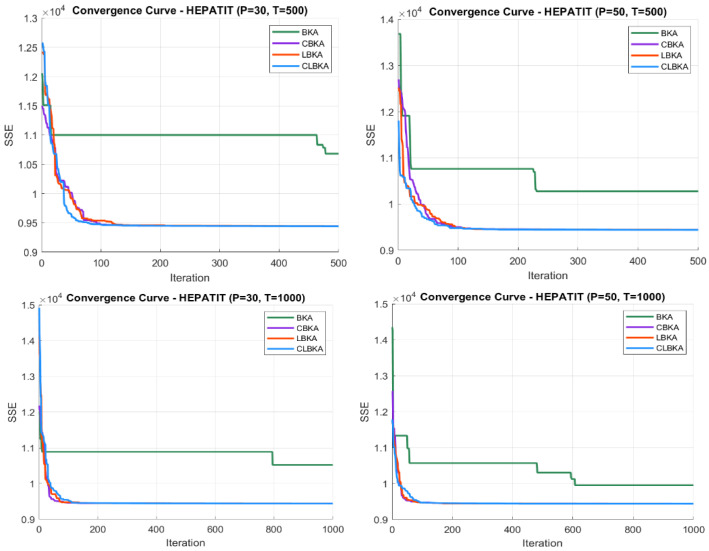
Convergence of the total square distance for **Hepatit** data set.

**Figure 19:**
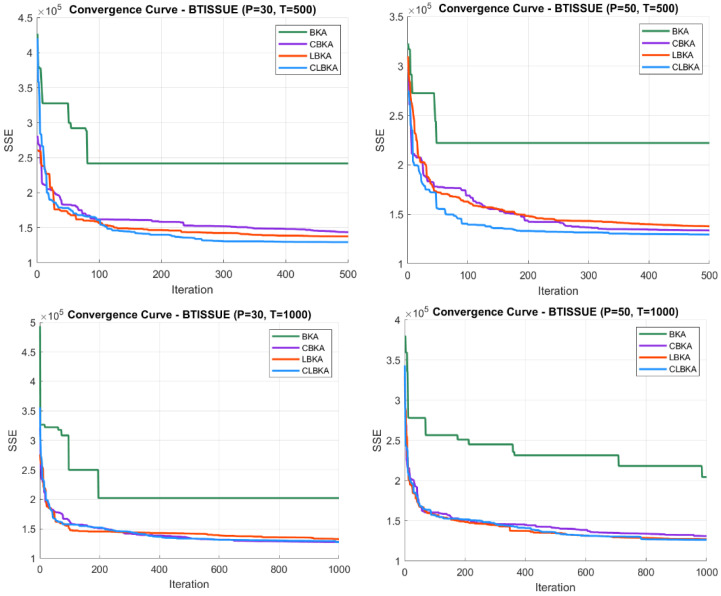
Convergence of the total square distance for **Btissue** data set.

**Figure 20:**
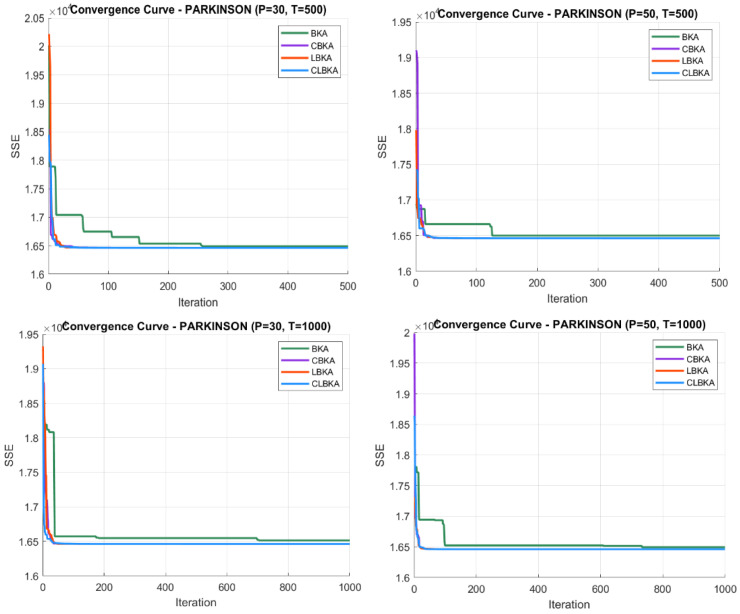
Convergence of the total square distance for **Parkinson** data set.

**Figure 21:**
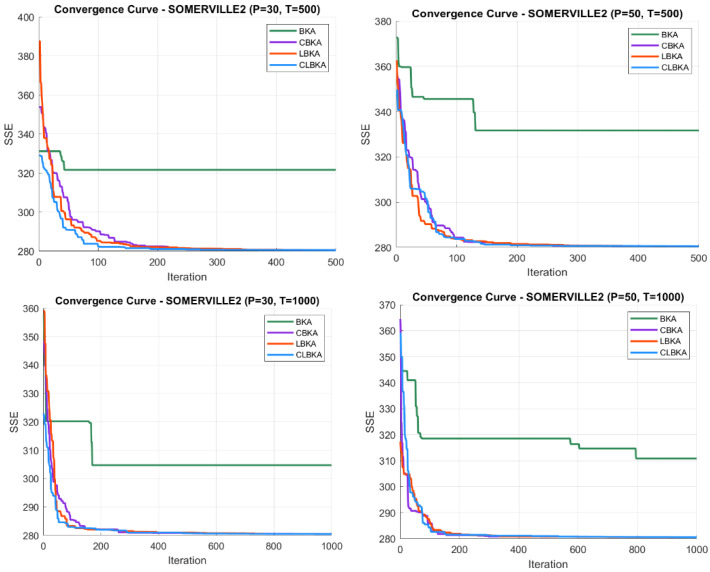
Convergence of the total square distance for **Somerville** data set.

Figure 7, Figure 8, Figure 9, Figure 10, Figure 11, Figure 12, Figure 13, Figure 14, Figure 15, Figure 16, Figure 17, Figure 18, Figure 19, Figure 20 and Figure 21 present the convergence behavior of all four algorithms across the evaluated datasets. Overall, CLBKA achieves faster and more stable convergence, typically reaching near-optimal solutions within the first few hundred iterations. While LBKA and CBKA show improved performance compared to BKA, their convergence is generally slower and less stable. Although CLBKA exhibits early stabilization in the convergence curves, this does not necessarily indicate harmful over-convergence. Rather, it reflects efficient descent toward high-quality solutions. In CLBKA, search diversity is preserved even in later iterations through chaotic parameter control, conditionally activated Lévy flights, and Cauchy-based migration perturbations. These mechanisms generate non-periodic variations and intermittent long-range steps, which prevent population stagnation and sustain adaptive search dynamics throughout the optimization process. The consistent performance across datasets of varying complexity confirms the robustness of this hybrid strategy.

### 4.2. Performance Evaluation of the Proposed Methods Against Literature-Reported Algorithms

Table 7 compares the proposed BKA variants with literature-reported algorithms under identical evaluation settings (population size = 30, iterations = 500). CLBKA consistently achieves top rankings on the majority of datasets, such as Balance, Credit, E. coli, Glass, Iris, Thyroid, Wine, Heart, Somerville, and User Modeling, demonstrating its robust and generalizable performance. However, CLBKA does not always outperform all competing methods. In the Spect dataset, PSO achieves the best SSE (537.339), suggesting that its strong local search capability better suits binary and overlapping data. In Dermatology, WOA performs best (2670.14), likely benefiting from balanced exploration–exploitation in high-dimensional spaces. The Thyroid dataset sees GWO as the top performer (1933.91), while in Diabetes, PSO again leads (49,269.24), possibly due to its convergence speed on noisy data. Additionally, GWO ranks first on B. Tissue (129,653), and PSO outperforms the others on Parkinson (12,363). These cases highlight that specific data characteristics, such as noise, feature distribution, or cluster shape, can affect algorithm suitability. Despite these instances, CLBKA remains the most consistently high-ranking approach across diverse benchmarks.

### 4.3. Statistical Evaluation via the Friedman Test

The Friedman test was employed to statistically compare the proposed methods across different population sizes and iteration budgets. As reported in Table 8, CLBKA consistently achieves the lowest Friedman mean rank in all parameter settings, indicating the best overall performance among the proposed approaches. LBKA and CBKA exhibit intermediate ranks that vary slightly across configurations, while BKA remains ranked last in every case, reflecting comparatively weaker clustering performance. The small variation in CLBKA’s mean rank values across settings further suggests that its superiority is robust and only weakly affected by changes in population size or iteration number. The Friedman test indicated statistically significant differences among the compared algorithms (*p* < 0.05).

**Figure 22:**
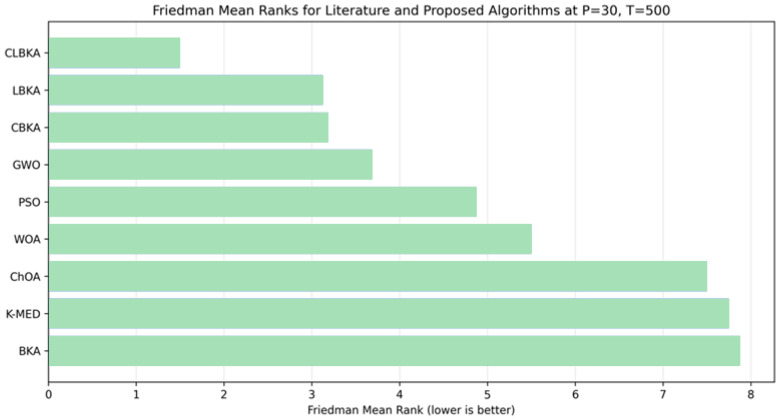
Friedman Mean Ranks for Literature and Proposed Algorithms.

Table 9 summarizes the Friedman mean-rank comparison between the literature-reported algorithms and the proposed methods under the benchmark setting with population size 30 and 500 iterations. CLBKA achieves the best overall rank under the same evaluation budget, followed by LBKA and CBKA as competitive alternatives. In contrast, BKA and several literature baselines yield higher mean ranks, indicating inferior overall performance. The distribution shown in Figure 22 further highlights the consistent advantage of CLBKA under matched experimental conditions.

### 4.4. Post Hoc Statistical Analysis Using the Nemenyi Test

Although the Friedman test provides a global indication of statistically significant differences among multiple algorithms, it does not identify which specific algorithm pairs differ significantly. Therefore, to strengthen the statistical analysis and comply with best practices in algorithm comparison, a Nemenyi post hoc test was conducted following the Friedman test.

The Nemenyi test compares all algorithm pairs based on their average ranks obtained from the Friedman test. The critical difference (*CD*) is calculated as:

$$CD=q_{\alpha }\cdot \sqrt{\frac{k(k+1)}{6N}}$$

where k  is the number of algorithms, N is the number of datasets, and $q_{\alpha }$ is the critical value of the Studentized range distribution. In this study, k=4  (BKA, LBKA, CBKA, and CLBKA), N=16  datasets, and $q_{0.05}=2.569\;$ for a significance level of α = 0.05. Substituting these values yields:

$$CD=2.569\cdot \sqrt{\frac{4(4+1)}{6\cdot 16}}\approx 1.17$$

Accordingly, any difference in average ranks greater than 1.17 is considered statistically significant. Using the Friedman mean ranks reported in Table 7, pairwise comparisons between CLBKA and the other algorithms were performed under all parameter settings. The results of the Nemenyi post hoc analysis are summarized in Table 10.

A post hoc Nemenyi test was conducted following the Friedman test to determine which algorithm pairs exhibit statistically significant differences. Based on 16 datasets and 4 algorithms, the critical difference (CD) at α = 0.05 was calculated as 1.17. Table 10 summarizes the pairwise comparisons using CLBKA as the reference. Results show that CLBKA significantly outperforms BKA and CBKA across all parameter settings. Although CLBKA often has better ranks than LBKA, the differences were not statistically significant in some configurations (P = 30, T = 1000 and P = 50, T = 1000), as the rank differences remained below the critical threshold. These results reinforce the superior performance of CLBKA and validate the statistical significance of its advantage under multiple experimental conditions.

### 4.5. Statistical Analysis Using the Wilcoxon Signed-Rank Test

The results of the Wilcoxon signed-rank test, presented in Table 9, Table 10, Table 11 and Table 12, evaluate the statistical significance of the performance differences between the proposed algorithms under various parameter settings. These pairwise comparisons provide deeper insight into whether the observed performance improvements of CLBKA are statistically meaningful or merely incidental.

As shown in Table 11, under the configuration P = 30 and T = 500, CLBKA significantly outperforms both CBKA and LBKA with very small *p*-values (*p* = 0.00006), and large negative effect sizes (r = −1.002), indicating strong and consistent superiority. Furthermore, BKA performs significantly worse than all three enhanced variants (*p* = 0.00044), confirming its relatively weaker optimization capability. On the other hand, the comparison between CBKA and LBKA yields a non-significant result (*p* = 0.89038), suggesting similar behavior between these two methods

As shown in Table 12, under the setting P = 30 and T = 1000, as shown in Table 10, the pattern remains consistent. CLBKA continues to significantly outperform CBKA (*p* = 0.00006) and LBKA (*p* = 0.00012), indicating that the hybrid strategy maintains its advantage even at higher iteration counts. The difference between CBKA and LBKA remains statistically non-significant (*p* = 0.09058), reinforcing the notion that these two algorithms are performance-wise comparable.

As reported in Table 13, when the population size increases to P = 50 while keeping T = 500, CLBKA retains its statistically significant superiority over both CBKA and LBKA (*p* = 0.00012 for both comparisons). BKA once again shows the weakest performance, significantly lagging behind all enhanced variants (*p* = 0.00044). These findings indicate that CLBKA remains effective even with a larger population, likely due to its balanced exploration–exploitation dynamics.

Finally, in Table 14 under the setting P = 50 and T = 1000, although *p*-values slightly increase, CLBKA still demonstrates significant performance advantages over CBKA (*p* = 0.00012, r = −0.960) and LBKA (*p* = 0.00159, r = −0.790). The performance gap between CBKA and LBKA remains statistically non-significant (*p* = 0.46484), which is consistent with earlier observations.

### 4.6. Sensitivity Analysis of the Lévy Exponent β

To examine the robustness of CLBKA with respect to its internal parameters, a comprehensive sensitivity analysis was conducted on the Lévy flight exponent β, which governs the step-size distribution in the global exploration phase. The β parameter was varied across two values (1.3 and 1.7), while all other parameters were kept constant. The analysis was carried out on 16 UCI benchmark datasets, and the results are summarized in Table 15. As observed, CLBKA demonstrates highly stable clustering performance under both β settings. The average SSE and Rand Index (RI) values show only minor fluctuations, suggesting that the algorithm is relatively insensitive to moderate changes in β. This stability further reinforces the practical reliability of CLBKA, especially in applications where parameter tuning is limited or computationally expensive.

## 5. Conclusions

This study examined the clustering performance of the Black-Winged Kite Algorithm and its enhanced variants, CBKA, LBKA, and CLBKA, developed to mitigate premature convergence and limited exploration capability observed in the standard BKA. Chaotic logistic mapping was employed to enhance population diversity and adaptive parameter regulation, while Lévy flight mechanisms improved long-range exploration. The hybrid CLBKA framework integrated these strategies with Cauchy-based perturbations to achieve a more balanced transition between exploration and exploitation during centroid optimization. The algorithms were evaluated on sixteen UCI benchmark datasets under different population sizes and iteration settings. Across all experimental configurations, CLBKA consistently achieved lower SSE values and improved convergence stability compared to the standard BKA and its single-enhanced variants. Statistical analyses using the Friedman and Wilcoxon tests confirmed significant performance differences, with CLBKA attaining the lowest mean rank across test conditions. Comparative evaluations against established metaheuristic clustering algorithms, including PSO, GWO, WOA, and ChOA, further demonstrated competitive and frequently superior performance across diverse datasets.

Despite these findings, several limitations should be acknowledged. The datasets considered are primarily small- to medium-scale, and the number of clusters was assumed to be known in advance. In addition, clustering performance was evaluated using the SSE objective function within a centroid-based framework relying on Euclidean distance, which may not be suitable for all data structures. Future research may extend this framework by incorporating alternative clustering objectives such as density-based, graph-based, or validity-index-driven optimization criteria, rather than relying solely on SSE minimization. Furthermore, large-scale implementations using parallel or GPU-based computation, automatic cluster number estimation, alternative distance metrics, and robustness improvements for noisy or imbalanced datasets represent promising research directions.

## Figures and Tables

**Table 1:** Summary of the characteristics of the datasets used in this study.

Dataset	Number of Cluster Centers	Number of Features	Number of Instances
**Balance**	3	4	625
**Credit**	2	14	690
**Dermatology**	6	34	366
**E. coli**	5	7	327
**Glass**	6	9	214
**Iris**	3	4	150
**Thyroid**	3	5	215
**Wine**	3	13	178
**Heart**	2	13	270
**Spect**	2	22	267
**Diabets**	2	8	768
**Hepatit**	2	21	155
**Btissue**	6	9	106
**Parkinson**	2	22	195
**Somerville**	2	6	143
**User Modeling**	4	5	258

**Table 7:** Performance comparison of the proposed methods with literature-reported clustering algorithms.

Dataset		K-MED [24]	TSA [68]	ChOA [36]	WOA [36]	PSO [36]	GWO [36]	BKA	LBKA	CBKA	CLBKA
**Balanc** **e**	A	1686.47	1502.1398	1452.44	1433.567	1426.63	1423.91	1447.66	1423.89	1423.88	**1423.87**
	Rank	10	9	8	6	5	4	7	3	2	**1**
**Credit**	A	562284	1653922	558675	566192	589789	556944	583138	556953	556982	**556834**
	Rank	6	10	5	7	9	2	8	3	4	**1**
**Dermatology**	A	2864.96	3410.7612	2842.06	2670.143	2708.78	**2459.79**	3182.72	2861.85	2860.77	2838.48
	Rank	8	10	5	2	3	**1**	9	7	6	4
**E. coli**	A	145.9961	138.9561	128.2704	84.104	80.478	74.878	115.568	72.2632	72.5915	**72.1794**
	Rank	10	9	8	6	5	4	7	2	3	**1**
**Glass**	A	311.0533	687.2979	494.8411	406.691	311.348	319.34	457.13	308.743	305.671	**299.149**
	Rank	4	10	9	7	5	6	8	3	2	**1**
**Iris**	A	183.6139	213.3086	147.6409	97.1151	110.990	99.265	148.97	96.8274	96.8728	**96.7764**
	Rank	9	10	7	4	6	5	8	2	3	**1**
**Thyroid**	A	2097.681	3929.0082	2490.993	2125.200	2178.346	1933.91	2691.15	1912.56	1908.09	**1899.24**
	Rank	5	10	8	6	7	4	9	3	2	**1**
**Wine**	A	17656.67	19588.19	16893.21	16450	16336	16328.8	16811.5	16326	16326.2	**16324.5**
	Rank	9	10	8	6	5	4	7	2	3	**1**
**Heart**	A	11814.20	12290.65	10514.37	9659.57	9497.74	9448.40	10314.4	9443.84	9443.74	**9443.25**
	Rank	9	10	8	6	5	4	7	3	2	**1**
**Spect**	A	633.544	659.8949	572.2234	562.972	**537.339**	556.563	620.493	557.417	558.056	556.296
	Rank	9	10	7	6	**1**	3	8	4	5	2
**Diabetes**	A	73172.07	93733.439	80797.25	72933.43	**49269.24**	72204.7	75681.3	72109.9	72173.7	72107.2
	Rank	7	10	9	6	**1**	5	8	3	4	2
**Hepatit**	A	10376.55	12471.312	10416.52	9571.687	9600.54	9447.17	10384.3	9443.82	9443.80	**9443.31**
	Rank	7	10	9	5	6	4	8	3	2	1
**B. Tissue**	A	143417	405517.18	153597	139204.3	186916	**129653**	239229	140130	137016	135735
	Rank	6	10	7	4	8	**1**	9	5	3	2
**Parkinson**	A	17120	18140	16527	16465	**12363**	16464	16547	16463	16463	16463
	Rank	7	8	5	4	**1**	3	6	2	2	2
**Somerville**	A	327.701	372.8451	316.421	283.599	287.471	280.654	318.476	280.567	280.579	**280.553**
	Rank	9	10	7	5	6	4	8	2	3	**1**
**User Modeling**	A	152.5859	122.9621	144.9154	104.874	99.988	98.844	113.159	98.2043	99.0395	**98.1158**
	Rank	10	8	9	6	5	3	7	2	4	**1**

**Table 8:** Friedman Mean Ranks of the Proposed Methods across Parameter Settings.

Parameter Setting	BKA	LBKA	CBKA	CLBKA
P = 30, T = 500	4.0000	2.4375	2.5000	**1.0625**
	4	2	3	**1**
P = 30, T = 1000	4.0000	2.1250	2.7812	**1.0938**
	4	2	3	**1**
P = 50, T = 500	4.0000	2.4062	2.4688	**1.1250**
	4	2	3	**1**
P = 50, T = 1000	4.0000	2.1562	2.5938	**1.2500**
	4	2	3	**1**

**Table 9:** Friedman Mean Ranks for Literature and Proposed Algorithms at P = 30, T = 500.

Parameter Setting	K-MED	ChOA	WOA	PSO	GWO	BKA	LBKA	CBKA	CLBKA
**P = 30, T** **= 500**	7.7500	7.5000	5.5000	4.8750	3.6875	7.8750	3.1250	3.1875	**1.5000**
	8	7	6	5	4	9	2	3	**1**

**Table 10:** Nemenyi post hoc comparisons using CLBKA as the reference algorithm.

Parameter Setting	BKA-CLBKA	LBKA-CLBKA	CBKA-CLBKA	Sig. Diff.
**P = 30,** **T = 500**	2.9375	1.3750	1.4375	BKA, LBKA, CBKA
**P = 30, T = 1000**	2.9062	1.0312	1.6874	BKA, CBKA
**P = 50, T = 500**	2.8750	1.2812	1.3438	BKA, LBKA, CBKA
**P = 50, T = 1000**	2.7500	0.9062	1.3438	BKA, CBKA

**Table 11:** Wilcoxon signed-rank test results for P = 30 and T = 500.

P = 30, T = 500			
Comparison	*p*-Value	z	r
**BKA** **vs. CBKA**	0.00044	3.516	0.879
**BKA vs. LBKA**	0.00044	3.516	0.879
**BKA vs. CLBKA**	0.00044	3.516	0.879
**CBKA vs. LBKA**	0.89038	−0.138	−0.034
**CLBKA vs. CBKA**	0.00006	−4.009	−1.002
**CLBKA vs. LBKA**	0.00006	−4.009	−1.002

**Table 12:** Wilcoxon signed-rank test results for P = 30 and T = 1000.

P = 30, T = 1000			
Comparison	*p*-Value	z	r
**BKA vs. CBKA**	0.00044	3.516	0.879
**BKA vs. LBKA**	0.00044	3.516	0.879
**BKA vs. CLBKA**	0.00044	3.516	0.879
**CBKA vs. LBKA**	0.09058	−1.692	−0.423
**CLBKA vs. CBKA**	0.00006	−4.009	−1.002
**CLBKA vs. LBKA**	0.00012	−3.842	−0.960

**Table 13:** Wilcoxon signed-rank test results for P = 50 and T = 500.

P = 50, T = 500			
Comparison	*p*-Value	z	r
**BKA vs. CBKA**	0.00044	3.516	0.879
**BKA vs. LBKA**	0.00044	3.516	0.879
**BKA vs. CLBKA**	0.00044	3.516	0.879
**CBKA vs. LBKA**	0.89258	−0.135	−0.034
**CLBKA vs. CBKA**	0.00012	−3.842	−0.960
**CLBKA vs. LBKA**	0.00012	−3.842	−0.960

**Table 14:** Wilcoxon signed-rank test results for P = 50 and T = 1000.

P = 50, T = 1000			
Comparison	*p*-Value	z	r
**BKA** **vs. CBKA**	0.00523	2.792	0.698
**BKA vs. LBKA**	0.00523	2.792	0.698
**BKA vs. CLBKA**	0.00523	2.792	0.698
**CBKA vs. LBKA**	0.46484	−0.731	−0.183
**CLBKA vs. CBKA**	0.00012	−3.842	−0.960
**CLBKA vs. LBKA**	0.00159	−3.158	−0.790

**Table 15:** Sensitivity analysis of CLBKA with respect to the Lévy exponent β.

P = 30, T = 500					
Dataset	β	AvgSSE	StdSSE	AvgRI	StdRI
**Balance**	1.3	**1423.8716**	0.013334	**0.587727**	0.007425
	1.7	1423.8721	**0.011012**	0.586405	**0.007357**
**Credit**	1.3	**556793**	**71.543694**	**0.524318**	**0**
	1.7	556836	120.51253	**0.524318**	**0**
**Dermatology**	1.3	**2840.290**	**22.934419**	**0.693953**	**0.007704**
	1.7	2864.921	27.135864	0.693186	0.008821
**E. coli**	1.3	71.66269	**1.273079**	**0.858257**	**0.019743**
	1.7	**71.45306**	1.296743	0.852987	0.025958
**Glass**	1.3	299.4440	6.579818	0.561745	0.018459
	1.7	**297.5927**	**5.202003**	**0.563213**	**0.016911**
**Iris**	1.3	**96.76454**	**0.036045**	**0.886198**	**0.002478**
	1.7	96.76530	0.036521	0.885183	0.002986
**Thyroid**	1.3	**1897.919**	12.66968	0.586284	**0.012291**
	1.7	1902.059	**12.02565**	**0.593960**	0.018926
**Wine**	1.3	**16324.09**	4.840715	0.725539	0.003500
	1.7	16324.46	**3.876592**	**0.726945**	**0.003347**
**Heart**	1.3	**9443.20**	**0.437336**	**0.570388**	2.26 × 10^−16^
	1.7	9443.32	0.521106	**0.570388**	**2.25 × 10^−16^**
**Spect**	1.3	**556.721**	0.856305	**0.674101**	3.9 × 10^−16^
	1.7	556.781	**0.716108**	**0.674101**	**3.3 × 10^−16^**
**Diabetes**	1.3	**72107.24**	**0.013673**	**0.546218**	**0**
	1.7	72107.25	0.018186	**0.546218**	**0**
**Hepatit**	1.3	9443.48	**0.486373**	**0.570388**	2.26 × 10^−16^
	1.7	**9443.36**	0.564837	**0.570388**	**2.25 × 10^−16^**
**B. Tissue**	1.3	136510.8	3632.838	**0.694662**	0.023726
	1.7	**136198.0**	**3065.630**	0.693646	**0.020872**
**Parkinson**	1.3	**16463.01**	0.028410	0.630769	1.13 × 10^−16^
	1.7	**16463.01**	**0.024071**	0.630769	**1.12 × 10^−16^**
**Somerville**	1.3	280.5583	**0.011305**	0.529597	0.005498
	1.7	**280.5567**	0.011742	**0.531164**	**0.004092**
**User Modeling**	1.3	**98.15417**	**0.444100**	**0.674856**	**0.009166**
	1.7	98.16058	0.455953	0.673770	0.010470

## Data Availability

The datasets used in this study were obtained from the UCI Machine Learning Repository and are publicly accessible. The relevant dataset links are cited in the References section. No new data were generated in this study.
